# Modeling Tracer Diffusion Coefficients of Any Type of Solutes in Polar and Non-Polar Dense Solvents

**DOI:** 10.3390/ma15186416

**Published:** 2022-09-15

**Authors:** Bruno Zêzere, Inês Portugal, José R. B. Gomes, Carlos M. Silva

**Affiliations:** CICECO–Aveiro Institute of Materials, Department of Chemistry, University of Aveiro, 3810-193 Aveiro, Portugal

**Keywords:** modeling, non-polar solvents, polar solvents, Rice and Gray, supercritical carbon dioxide, tracer diffusion coefficients, transport properties, water

## Abstract

In this work, a simple two-parameters correlation based on the Rice and Gray, Lennard-Jones, and Stockmayer theories was devised for the calculation of binary diffusion coefficients ($D_{12}$) of any type of solutes at infinite dilution in polar and non-polar solvents. This equation can be relevant for systems with polar solvents, since most models in the literature fail when strong intermolecular forces predominate in solution. The new correlation embodies the Stockmayer potential without requiring the dipole moments of any component, which significantly enlarges its application. It was validated with the largest $D_{12}$ database of polar and non-polar dense systems, with 8812 data points (NDP) spanning 553 systems, of which 133 have water as solvent (NDP = 1266), 89 contain polar solvents excluding water (NDP = 1405), 177 have supercritical carbon dioxide (SC-CO2) as solvent (NDP = 5028), and 154 have non-polar or weakly polar solvents excluding SC-CO2 (NDP = 1113). Overall, the model achieved an average deviation of only 3.43%, with accurate and unbiased behavior even for polar systems.

## 1. Introduction

Diffusion coefficients, namely binary diffusivities ($D_{12}$), are of major importance for the chemical and related industries since it is a transport property required for the accurate design and simulation of processes limited by mass transfer kinetics [1]. The experimental measurement of $D_{12}$ is expensive in terms of equipment, chemicals, time, and the necessary apparatuses are frequently limited by their intrinsic range of operation that cannot encompass wide intervals of temperature and pressure. Hence, accurate models applicable over a wide range of temperature, pressure, and solvent and solute types (both in terms of size, molecular weight, chemical nature, and polarity) are of utmost importance.

For nonpolar and weekly polar solvent systems, which may or may not include polar solutes, there are already several published models providing good results. In 2021 Zêzere et al. [2] compared various models using a database containing 6180 data points of weakly and non-polar systems, and expressed their performances in terms of the well-known average absolute relative deviation (AARD):

(1) $$\mathrm{AARD}\;\left(\%\right)=\frac{100}{\mathrm{NDP}}\overset{\mathrm{NDP}}{\underset{i=1}{\sum }}\left|\frac{D_{12,i}^{\mathrm{calc}}-D_{12,i}^{\exp}}{D_{12,i}^{\exp}}\right|$$

where NDP is the number of data points, and the superscripts calc and exp stand for calculated and experimental, respectively. The hydrodynamic Wilke-Chang equation [3], one of the simplest $D_{12}$ models, achieved good predictive results with AARD = 15.64%. Additionally, the predictive hybrid free-volume model of Tracer Liu-Silva-Macedo (TLSM) [4,5] attained an equivalent AARD of 16.84%, while their derived 1-parameter TLSM_d_ and TLSM_en_ correlations achieved average deviations of 4.53% and 4.55%, respectively, which represent a significant improvement over the seminal equation. Finally, the 2-parameters free-volume model of Dymond–Hildebrand–Batschinski (DHB) [6,7] achieved AARD = 4.23%; it is the simplest equation in the literature, and can be linearized to obtain parameters from experimental data.

On the other hand, in the case of polar solvents the situation is quite different with fewer models being applicable due to the increased complexity of the intermolecular interactions, mainly hydrogen bonding [8,9]. For instance, the predictive Wilke-Chang and Tyn-Calus equations [10] reached high deviations (AARD = 30.05% and 28.96%, respectively) when tested with 1994 data points from polar systems [11]. Concerning correlations, the DHB model (2 parameters, AARD = 6.02%) and the Rice and Gray approach of Magalhães et al. [11] (2 parameters, AARD = 4.27%) obtained good results for the same database [11]. Nonetheless, the last correlation requires the knowledge of the dipole moments of both solute and solvent which are frequently non-available or very complex to estimate and, unlike this essay, has been validated with a much smaller database (3463 data points/211 binary systems, from which 1994 data points/141 systems are for polar solvents). Finally, a machine learning approach based on the gradient boost model was developed by Aniceto et al. [12] achieving reliable results (AARD = 5.07%) especially in comparison with the previously mentioned equations.

Various empirical equations are available and can be applied to polar and non-polar solvent systems, namely those proposed by Magalhães et al. [13] and others sparsely tested in the literature [14,15,16,17,18]. Notwithstanding their suitability for $D_{12}$ correlation and the good results achieved (e.g., Magalhães et al. [13] obtained AARD = 2.8% for 8219 data points/539 systems), their scope of application is difficult to assess and they are generally tested with few data points (with exception for the work of Magalhães et al.).

In this work an upgraded Rice and Gray-based correlation for the calculation of binary diffusivities is presented and, with the objective to extend its applicability to polar solvent systems, a combined Stockmayer and Lennard-Jones (LJ) potential term is embodied. Unlike a previous approach in the field [11], the proposed model does not require the solvent molar volume at normal boiling point and the dipole moments of both solute and solvent which expands its applicability to any type of solutes in supercritical fluids, liquids, and dense gases involving nonpolar, weakly polar, and polar solvents. The model is tested with the largest database compiled until now comprehending 8812 data points (NDP) from 553 systems, from which 2671 points are from systems with a polar solvent, including water, and the remaining 6141 points are from nonpolar or weakly polar solvent systems where supercritical carbon dioxide (SC-CO_2_) is included. In relation to the previous work on polar systems [11] this essay includes a much larger database for validation, namely, 5349 (=8812–3463) points of which 677 (=2671–1994) are specific for polar solvents. 

## 2. Model Development and Database

### 2.1. Rice and Gray $D_{12}$ Correlation

The $D_{12}$ model is based on the general Einstein equation [19,20], which relates diffusivity (D) with absolute temperature (T) and a non-null friction coefficient (ξ):

(2) $$=\frac{k_{B}T}{\xi }=\frac{k_{B}T}{\xi^{H}+\xi^{S}}=\frac{k_{B}T}{\xi^{H}+\xi^{S,S}+\xi^{S,P}}$$

where $k_{B}$ is the Boltzmann constant ($1.380658\times 10^{-16}\;g\;{\mathrm{cm}}^{2}{\;s}^{-2}\;K$), ξ is the sum of a hard core ($\xi^{H}$) and an attractive ($\xi^{S}$) contribution, and $\xi^{S}$ embodies a Lennard-Jones (LJ) ($\xi^{S,S}$) and a polar ($\xi^{S,P}$) term. The $\xi^{H}$ coefficient is expressed by modifying the Enskog equation [21,22] with a correction term F [1]:

(3) $$\xi^{H}=\frac{8}{3}\;\rho_{n}\;\sigma_{\;}{}^{2}\;\sqrt{2\pi mk_{B}\;T\;}\frac{g\left(\sigma \right)}{F}$$

where $\rho_{n}$ is the number density, σ is the molecule diameter, m is its mass, $g\left(\sigma \right)$ is the radial distribution function at contact, and F is the hard sphere correction factor. The $\xi^{S,S}$ is given by the recipe employed by Ruckenstein and Liu [23], and $\xi^{S,P}$ is proposed embodying the method of Brokaw [24]:

(4) $$\xi^{S,S}=\frac{8}{3}\;\rho_{n}\;\sigma_{\;}{}^{2}\;\sqrt{2\pi \;m_{\;}k_{B}\;T\;}\frac{0.4}{T^{*}{}^{1.5}}$$

(5) $$\xi^{S,P}=\frac{8}{3}\;\rho_{n}\;\sigma_{\;}{}^{2}\;\sqrt{2\pi \;m_{\;}k_{B}\;T\;}\frac{\delta_{\;}{}^{2}}{T^{*}{}^{1.5}}$$

where $T_{\;}^{*}$ is the reduced temperature and δ the Stockmayer parameter [24]. The sum of $\xi^{S,S}$ and $\xi^{S,P}$ is now expressed in terms of a fitting coefficient $B=0.4+\delta^{2}$:

(6) $$\xi^{S,S}+\xi^{S,P}=\frac{8}{3}\;\rho_{n}\;\sigma_{\;}{}^{2}\;\sqrt{2\pi \;m_{\;}k_{B}\;T\;}\frac{B}{T_{\;}^{*}{}^{1.5}}$$

In this way, the Stockmayer parameter is eliminated from Equation (6) and the dipole moments are no longer required for the δ calculation, in opposition to the original work of Magalhães et al. [11]. This approach expands the model applicability, since dipole moments are not frequently available and their accurate estimation is not easy. This is especially true in systems with complex molecules, such as, for instance, astaxanthin and quercetin [25,26] that, in the case of the latter, exhibits 48 possible stable conformers with dipole moments varying from 0.35 to 9.87 Debye under vacuum [27].

The unary quantities of the previous expressions should be now replaced by the corresponding binary ones, giving rise to the core equation:

(7) $$D_{12}^{\;}=\frac{k_{B}\;T}{\frac{8}{3}\;\rho_{n,1}\;\sigma_{\mathrm{eff},12}{}^{2}\;\sqrt{2\pi \;m_{12\;}k_{B}\;T\;}\left[\frac{g\left(\sigma_{\mathrm{eff},12}\right)}{F_{12}}+\frac{B_{12}}{T_{12}^{*}{}^{1.5}}\right]}$$

where subscripts 1 and 2 denote solvent and solute, respectively, $\rho_{n,1}$ is the number density of the solvent, $\sigma_{12,\mathrm{eff}}$ is the binary effective hard sphere diameter (where the Ben–Amotz–Herschbach expression [28] adopted for the *effective* calculation), $m_{12}$ is the reduced mass of the system, $g\left(\sigma_{\mathrm{eff},12}\right)$ is the pair radial distribution function at contact [29], $F_{12}$ is the hard sphere correction factor for $D_{12}^{\;}$ [30] and $T_{12}^{*}$ is the binary reduced temperature. For the calculation of the above-mentioned properties it is further required to know the critical volume ($V_{c}$), the critical temperature ($T_{c}$), and the mass (m) of both solute and solvent, and the mass density of the solvent ($\rho_{1}$) at the desired temperature and pressure. Knowing the properties of the pure compounds one can estimate the previously mentioned properties by recurring to Equations (8)–(19),

(8) $$\sigma_{\mathrm{LJ},j}\left(Å\right)=0.7889\times 10^{-8\;}V_{c,j}{\left({\mathrm{cm}}^{3}{\;\mathrm{mol}}^{-1}\right)}^{1/3},\;\;\;j=1\;\mathrm{or}\;2$$

(9) $$\frac{\varepsilon_{\mathrm{LJ},j}}{k_{B}}=\frac{T_{c,j}\left(K\right)}{1.2593}\;,\;\;\;j=1\;\mathrm{or}\;2$$

(10) $$\sigma_{\mathrm{LJ},12}=\left(1-k_{12}\right)\frac{\sigma_{\mathrm{LJ},1}+\sigma_{\mathrm{LJ},2}}{2}$$

(11) $$\frac{\varepsilon_{\mathrm{LJ},12}}{k_{B}}=\sqrt{\frac{\varepsilon_{\mathrm{LJ},1}}{k_{B}}\frac{\varepsilon_{\mathrm{LJ},2}}{k_{B}}}$$

(12) $$T_{j}^{*}=\frac{T}{\varepsilon_{\mathrm{LJ},j}/k_{B}},j=1,\;2\;\mathrm{or}\;12$$

(13) $$\sigma_{\mathrm{eff},j}=1.1532\;\sigma_{\mathrm{LJ},j}{\left(1+\sqrt{1.8975\;T_{j}^{*}}\right)}^{-1/6},j=1,\;2\;\mathrm{or}\;12$$

(14) $$\rho_{n,1}^{*}=\rho_{n,1}\;\sigma_{\mathrm{eff},1}{}^{3}$$

(15) $$\varphi =\frac{\pi }{6}\rho_{n,1}^{*}$$

(16) $$g\left(\sigma_{\mathrm{eff},12}\right)=\frac{1}{{\left(1-\varphi \right)}^{3}}\left(1-\varphi +\frac{2\varphi }{1+\sigma_{\mathrm{eff},1}/\sigma_{\mathrm{eff},2}}\right)\left(1-\varphi +\frac{\varphi }{1+\sigma_{\mathrm{eff},1}/\sigma_{\mathrm{eff},2}}\right)$$

(17) $$F_{11}=1+0.94605\rho_{n,1}^{*}{}^{1.5}+1.4022\rho_{n,1}^{*}{}^{3}-5.6898\rho_{n,1}^{*}{}^{5}+2.6626\rho_{n,1}^{*}{}^{7}$$

(18) $$F_{12}=\frac{F_{11}+\rho_{n,1}^{*}{}^{1.7}\left(a\ln\left(\sigma_{\mathrm{eff},2}/\sigma_{\mathrm{eff},1}\right)+b\ln{\left(\sigma_{\mathrm{eff},2}/\sigma_{\mathrm{eff},1}\right)}^{2}+c\ln\left(m_{2}/m_{1}\right)\right)}{1+\rho_{n,1}^{*}{}^{3}{\left(d\ln\left(\sigma_{\mathrm{eff},2}/\sigma_{\mathrm{eff},1}\right)\right)}^{2}}\;\mathrm{with}:\;\left\{\begin{array}{c} a=-1.676382\;\rho_{n,1}^{*}+1.638561\\ b=-8.516830\;\rho_{n,1}^{*}+8.631536\\ c=-1.320347\;\rho_{n,1}^{*}+1.351067\\ d=-5.062546\;\rho_{n,1}^{*}+5.409662 \end{array}\right.$$

(19) $$m_{12}=\frac{m_{1}m_{2}}{m_{1}+m_{2}}$$

where $\sigma_{\mathrm{LJ},j}$ is the LJ diameter of component j [23], $\varepsilon_{\mathrm{LJ},j}/k_{B}$ is the LJ energy parameter of j [23], $\sigma_{\mathrm{LJ},12}$ and $\varepsilon_{\mathrm{LJ},12}/k_{B}$ are the binary LJ parameters, $T_{j}^{*}$ is the reduced temperature of j, $\sigma_{\mathrm{eff},j}$ is the effective hard sphere diameter of component j calculated by the Ben–Amotz–Herschbach expression [28], $\rho_{n,1}^{*}$ is the reduced number density of the solvent, φ is the packing fraction of the solvent and $F_{11}$ is the correction factor for the self-diffusion coefficient of the solvent [23].

Besides the parameter $B_{12}$ explicit in Equation (7), a second fitting constant is now introduced in the model, namely, the binary interaction parameter $k_{12}$ embodied in the Lennard-Jones diameter combining rule (Equation (10)).

### 2.2. Parameters Optimization and Model Assessment 

**Figure 1:**
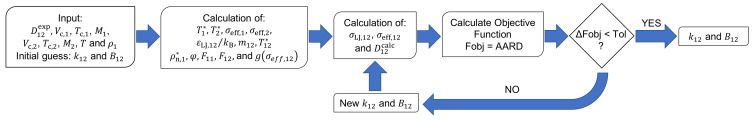
Schematic representation of the optimization process. The objective function is in this work the AARD (Equation (1)).

The optimization of the $D_{12}$ parameters ($k_{12}$ and $B_{12}$) was performed using the Nelder-Mead simplex algorithm described in Lagarias et al. [31] and codified in the *fminsearch* function of Matlab R2017b [32], adopting AARD (Equation (1)) as objective function. The model performance was assessed in terms of AARD (%) and average relative deviation (ARD, %, Equation (20)). A flowchart of the optimization procedure can be observed in Figure 1.

(20) $$\mathrm{ARD}=\frac{100}{\mathrm{NDP}}\overset{\mathrm{NDP}}{\underset{i=1}{\sum }}\frac{D_{12,i}^{\mathrm{calc}}-D_{12,i}^{\exp}}{D_{12,i}^{\exp}}$$

### 2.3. Database and Compounds Properties

The database utilized in this work consists of a total of 8812 data points from 553 systems which were divided into four subsets: (i) 177 systems with supercritical carbon dioxide SC-CO_2_ as solvent (NDP = 5028); (ii) 154 systems with nonpolar or weakly polar solvents excluding SC-CO_2_ (NDP = 1113); (iii) 88 systems with polar solvents excluding water (NDP = 1405); and (iv) 133 systems with water as solvent (NDP = 1266). All the diffusion data were taken from [15,16,17,18,25,26,33,34,35,36,37,38,39,40,41,42,43,44,45,46,47,48,49,50,51,52,53,54,55,56,57,58,59,60,61,62,63,64,65,66,67,68,69,70,71,72,73,74,75,76,77,78,79,80,81,82,83,84,85,86,87,88,89,90,91,92,93,94,95,96,97,98,99,100,101,102,103,104,105,106,107,108,109,110,111,112,113,114,115,116,117,118,119,120,121,122,123,124,125,126,127,128,129,130,131,132,133,134,135,136,137,138,139,140,141,142,143,144,145,146,147,148,149,150,151,152,153,154,155,156,157,158,159,160,161,162,163,164,165,166,167,168,169,170,171,172,173,174,175,176,177,178,179,180,181,182,183,184,185,186,187,188,189,190,191,192,193,194,195,196,197,198,199,200,201,202,203,204,205,206,207,208,209,210,211,212,213,214,215,216,217,218,219,220,221,222,223,224,225,226,227,228,229,230,231,232,233,234,235,236,237,238].

The properties required for modeling, whenever not reported in the original articles, were calculated or retrieved from the literature. In general, densities and viscosities were taken from the NIST database [239] or calculated by appropriate equations from Yaws [240], Cibulka and Ziková [241], Cibulka et al. [242,243], Cibulka and Takagi [244], Przezdziecki and Sridhar [8], Viswanath et al. [245], Lucas [245,246], Assael et al. [247], Cano-Gómez et al. [248], and Pádua et al. [249]. For the particular case of SC-CO_2_ densities and viscosities were estimated by the correlations of Pitzer and Schreiber [250] and Altunin and Sakhabetdinov [251], respectively, and for water they were obtained from IAPWS-IF97 [252]. The properties of the pure compounds (i.e., molecular weight ($M_{j}$), critical temperature ($T_{c,j}$), and critical volume ($V_{c,j}$)) are provided in Supplementary Material (Table S1).

In general, the fitting results achieved were good. However, some systems/data points were excluded from the database because the data were outside the domain of application of the equations used to estimate $F_{11}$ and/or $F_{12}$ delivering negative values without physical meaning. This was the case for carbon disulfide [161], methanol [161] and 1-alkyl-3-methylimidazolium ionic liquids (i.e., [Bmim][bti], [Emim][bti], [Hmim][bti] and [Omim][bti]) [160] in acetonitrile, and for 9,10-dimethylanthracene in *n*-hexane at 298.15 K and 3500 bar [149]. Nevertheless, the exclusion of these points corresponds to only 0.52% of the eligible compiled data.

## 3. Results and Discussion

The model performance was evaluated in terms of AARD and ARD. The global results achieved by the model and the results for each subset of systems are presented in Table 1. The individual deviations obtained for each system are provided in Supplementary Material (Tables S2–S5).

**Figure 2:**
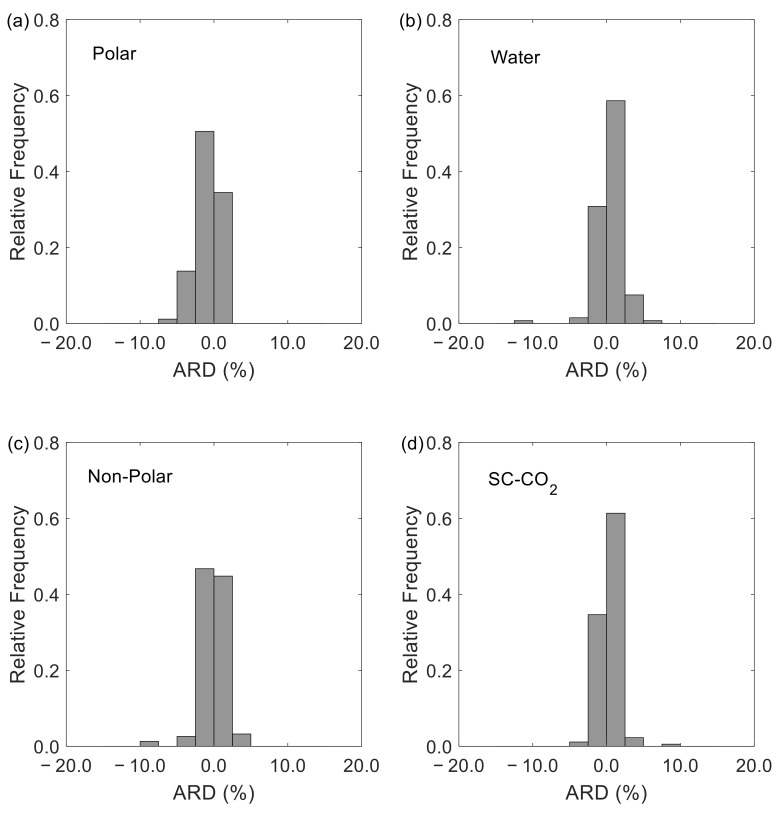
ARD histograms with bin sizes of 2.5% for systems with: (**a**) polar solvents, excluding water; (**b**) water as solvent; (**c**) non-polar and weakly polar solvents, excluding SC-CO_2_; and (**d**) SC-CO_2_ as solvent.

Overall, the $D_{12}$ model offers excellent results with very low AARD and ARD values as evidenced in Table 1 and shown graphically in Figure 2. Altogether, for the 553 studied systems the global AARD is 3.43% and the global ARD is 0.13%. For the four subsets analyzed the AARD values are within 3.23% and 3.96%. As for the individual systems, the top three with the highest deviations correspond to carbon tetrabromide (CBr_4_) in *n*-hexane [149], pyrene (C_16_H_10_) in *n*-hexane [149], and potassium chloride (KCl) in water [97,100,117], with AARD values of 22.48%, 21.89% and 15.26%, respectively.

Generally, no bias was observed in any subset of systems as demonstrated by the ARD values of −0.36%, −0.31%, 0.16%, and 0.35% for the polar, non-polar, water, and SC-CO_2_ systems, respectively. These observations are further highlighted in Figure 1 where one confirms that only three systems exhibit ARD values above 10% or below −10%. Interestingly, these systems are the same as those mentioned above (in the AARD analysis) and present ARD values of −16.94%, −18.07% and −11.15%.

**Figure 3:**
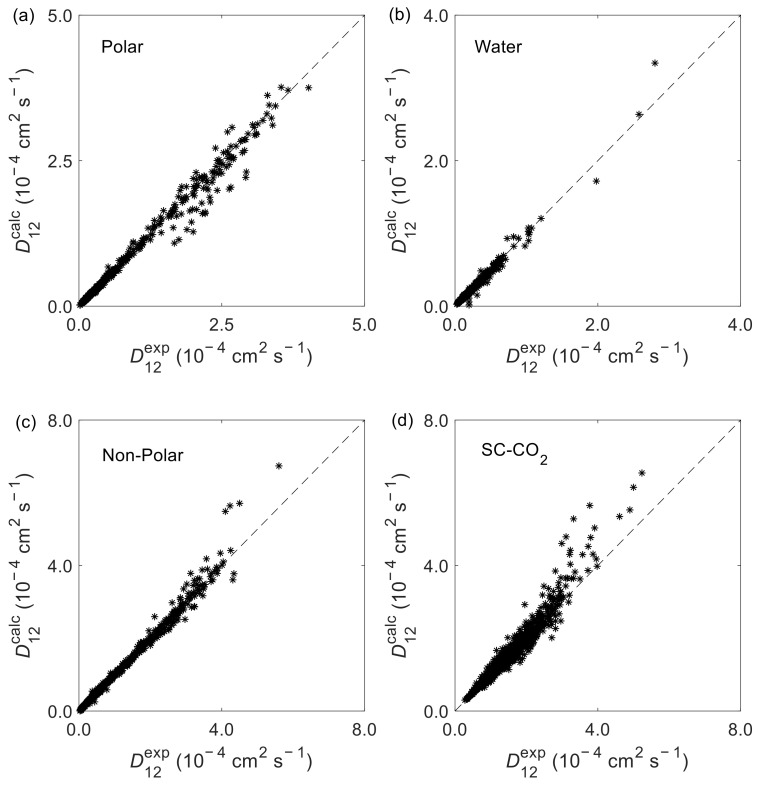
Calculated ($D_{12}^{\mathrm{calc}}$) vs. experimental ($D_{12}^{\exp}$ ) binary diffusion coefficients for systems with: (**a**) polar solvents, excluding water; (**b**) water as solvent; (**c**) non-polar and weakly polar solvents, excluding SC-CO_2_; and (**d**) SC-CO_2_ as solvent.

**Figure 4:**
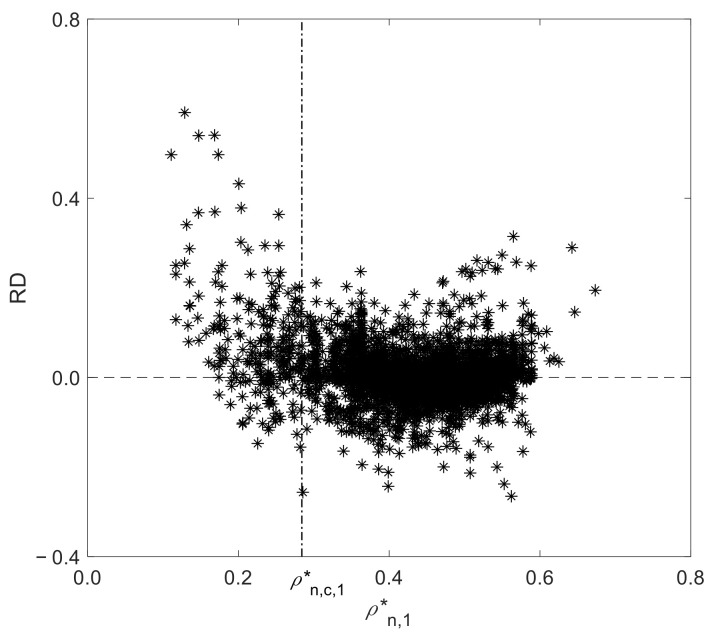
Relative deviation (RD) vs. reduced number density ($\rho_{n,1}^{*}$) of the SC-CO_2_ diffusivity subset. The vertical dashed line identifies the reduced number density of CO_2_ ($\rho_{n,1,c}^{*})$.

The calculated vs. experimental $D_{12}$ results illustrated in Figure 3 corroborate the previous AARD and ARD analysis, evidencing the very good performance of the $D_{12}$ model. The higher deviations observed for the SC-CO_2_ subset are further analyzed in Figure 4, where the relative deviations (RD) against the reduced number density of solvent, $\rho_{n,1}^{*}$, are plotted. As illustrated, the higher deviations tend to be found closer and below the critical density where accurate $D_{12}$ measurements are frequently difficult to carry out as well as their modeling; hence, such deviations may be expected in advance.

**Figure 5:**
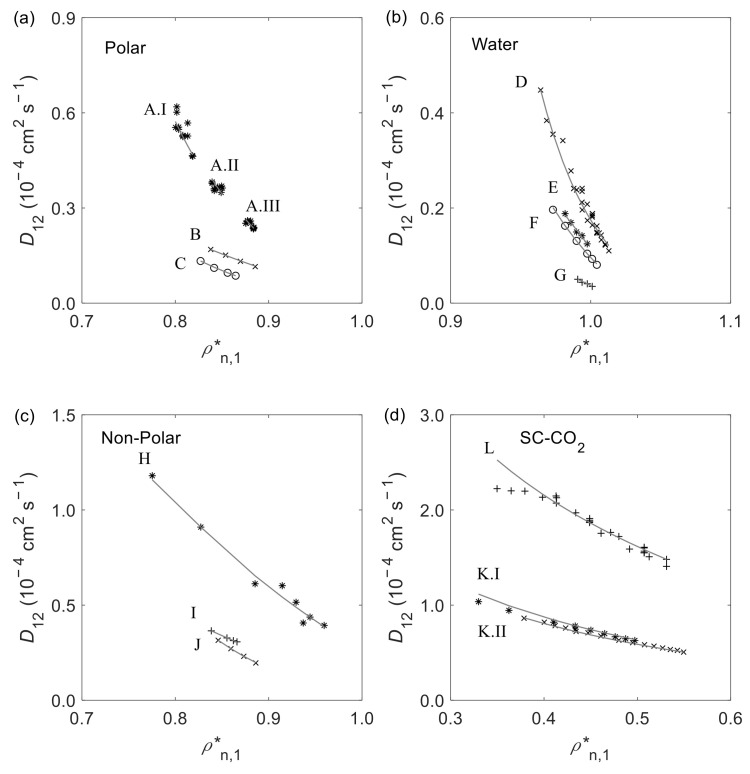
Representation of experimental (mark) and predicted (line) $D_{12}$ values vs. reduced number density of the solvent ($\rho_{n,1}^{*}$), at fixed T or P, for systems with: (**a**) polar solvents excluding water; (**b**) water as solvent; (**c**) non-polar and weakly polar solvents, excluding SC-CO_2_; and (**d**) SC-CO_2_ as solvent. The letters identify the following systems: A—ethanol/propane at 348.15 K (I), 323.15 K (II), and 298.15 K (III); B—ethyl acetate/quercetin at 1 bar; C—ethanol/palladium(II) acetylacetonate at 1 bar; D—water/methane at 1 bar; E—water/[Bmim][PF6] at 1 bar; F—water/alanine at 1 bar; G—water/*α*-cyclodextrin at 1 bar; H—*n*-octane/xenon at 1 bar; I—*n*-hexane/ferrocene at 313.15 K; J—cyclohexane/acetone at 160 bar; K—SC-CO_2_/eicosapentaenoic acid at 333.15 K (I) and 313.15 K (II); L—SC-CO_2_/ethanol at 313.21 K.

The experimental and calculated $D_{12}$ vs. $\rho_{n,1}^{*}$ is presented in Figure 5 for various solutes and solvents at fixed temperature and pressure. In general, the results are accurately described by the new $D_{12}$ model. For the polar systems shown in Figure 5a (i.e., ethanol/propane (A) at 298.15 K, 323.15 K, and 348.15 K [217], ethyl acetate/quercetin (B) at 1 bar [25], and ethanol/palladium(II) acetylacetonate (C) at 1 bar [121]) an excellent representation can be observed especially for systems B and C. Regarding the subset of water as solvent (see Figure 5b), the comparison of the experimental and calculated diffusivities of methane (D) [53,54], ionic liquid [Bmim][PF6] (E) [45], alanine (F) [49], and α-cyclodextrin (G) [95], all at P = 1 bar, clearly emphasizes the wide range of applicability of the model. For the subset of non-polar and weakly polar solvents excluding SC-CO_2_ (see Figure 5c), it is observed that the model predicts $D_{12}$ for diverse systems and conditions, including *n*-octane/xenon (H) at 1 bar [125], *n*-hexane/ferrocene (I) at 313.15 K [122], and cyclohexane/acetone (J) at 1 bar and 160 bar [118]. Finally, Figure 5d illustrates the results for SC-CO_2_ as solvent, namely for ethanol (L) at 313.21 K [190] and eicosapentaenoic acid (K) at 313.15 K and 333.15 K [227], respectively. Notwithstanding the good results achieved for most systems, higher deviations are observed for ethanol at low CO_2_ density which correspond to $D_{12}$ measurements close to the critical point (see previous comment). Ultimately, it may be concluded that the $D_{12}$ model has a wide range of applicability enabling the calculation of diffusion coefficients of simple (e.g., monoatomic species such as xenon) and complex (e.g., α-cyclodextrin and ionic liquids such as [Bmim][PF6]) solutes in polar or non-polar dense solvents, under isothermal or isobaric conditions.

A program that allows the estimation of diffusivities using the present model can be found in the Supplementary Material along with instructions on its use.

## 4. Conclusions

In this work, an upgraded Rice and Gray-based correlation is proposed for the calculation of binary diffusivities of solutes in polar and non-polar solvents. The model embodies a combined and simplified Stockmeyer and Lennard-Jones potential contribution in $D_{12}$ but does not require the dipole moments of either solute or solvent. Moreover, the solvent normal boiling point and solvent molar volume at normal boiling point are also not necessary. Hence, the applicability of the new correlation is significantly extended in terms of chemical species. 

The model validation was accomplished using the literature data for 553 polar and non-polar systems (8812 data points) involving diverse solvents and solutes. Overall, the model achieves very low deviations, with AARD = 3.43% and ARD = 0.13%, suggesting a consistent unbiased behavior. The database was divided into four subsets according to the nature of the solvent, namely polar solvents (excluding water), water, and non-polar and weakly polar solvents (excluding SC-CO_2_), and SC-CO_2_. Excellent results were obtained for all subsets with the exception of the low-density SC-CO_2_ region, i.e., near the critical point.

## Figures and Tables

**Table 1:** Performance of the $D_{12}$ model for each subset of systems and for the global database.

Solvent	Polar	Water	Non-Polar	SC-CO_2_	Global
Nsys	89	133	154	177	553
NDP	1405	1266	1113	5028	8812
AARD (%)	3.77	3.96	3.29	3.23	3.43
ARD (%)	−0.36	0.16	−0.31	0.35	0.13

Nsys—number of systems; NDP—number of data points.

## Data Availability

No new data were created or analyzed in this study. Data sharing is not applicable to this article.

## Supplementary Materials

The following supporting information can be downloaded at: https://www.mdpi.com/article/10.3390/ma15186416/s1: Software; Table S1. Pure component data, compound name, chemical formula, CAS number, molecular weight, M, critical temperature, $T_{c}$, and volume, $V_{c}$; Table S2. Polar solvents systems studied, number of data point (NDP), reduced number density range ($\rho_{n,1}^{*}$ range), absolute temperature range (T range), binary interaction parameters ($k_{12}$ and $B_{12}$), average absolute relative deviations (AARD), and source of the diffusion data; Table S3. Water solvent systems studied, number of data point (NDP), reduced number density range ($\rho_{n,1}^{*}$ range), absolute temperature range (T range), binary interaction parameters ($k_{12}$ and $B_{12}$), average absolute relative deviations (AARD), and source of the diffusion data; Table S4. Non-polar solvent systems studied, number of data point (NDP), reduced number density range ($\rho_{n,1}^{*}$ range), absolute temperature range (T range), binary interaction parameters ($k_{12}$ and $B_{12}$), average absolute relative deviations (AARD), and source of the diffusion data; Table S5. SC-CO_2_ solvent systems studied, number of data point (NDP), reduced number density range ($\rho_{n,1}^{*}$ range), absolute temperature range (T range), binary interaction parameters ($k_{12}$ and $B_{12}$), average absolute relative deviations (AARD), and source of the diffusion data.
