# Supplementary material for: Modeling Tracer Diffusion Coefficients of Any Type of Solutes in Polar and Non-Polar Dense Solvents
Source: Materials (Basel). 2022 Sep 15;15(18):6416. doi: 10.3390/ma15186416 (PMC9502877; doi:10.3390/ma15186416)
Supplement: Supplementary file 1 [file materials-15-06416-s001.zip › materials-1882225-supplementary.pdf]

Supplementary Material

Modeling tracer diffusion coefficients of any type of solutes in polar and non-polar dense solvents

Bruno Zêzere, Inês Portugal, José R. B Gomes, Carlos M. Silva*

CICECO – Aveiro Institute of Materials, Department of Chemistry, University of Aveiro, 3810-193 Aveiro, Portugal

*Correspondence: carlos.manuel@ua.pt

Software 2

Table S1. Pure component data, compound name, chemical formula, CAS number, molecular weight, M, critical temperature, T_c, and volume, V_c. 2

Table S2. Polar solvents systems studied, number of data point (NDP), reduced number density range ($\rho_{n,1}^*$ range), absolute temperature range (T range), binary interaction parameters (k_{12} and B_{12}), average absolute relative deviations (AARD) and source of the diffusion data. ...12

Table S3. Water solvent systems studied, number of data point (NDP), reduced number density range ($\rho_{n,1}^*$ range), absolute temperature range (T range), binary interaction parameters (k_{12} and B_{12}), average absolute relative deviations (AARD) and source of the diffusion data. ...17

Table S4. Non-polar solvent systems studied, number of data point (NDP), reduced number density range ($\rho_{n,1}^*$ range), absolute temperature range (T range), binary interaction parameters (k_{12} and B_{12}), average absolute relative deviations (AARD) and source of the diffusion data. ...23

Table S5. SC-CO_2 solvent systems studied, number of data point (NDP), reduced number density range ($\rho_{n,1}^*$ range), absolute temperature range (T range), binary interaction parameters (k_{12} and B_{12}), average absolute relative deviations (AARD) and source of the diffusion data. ...30

References 38

Software

The model developed in this work is provided as a Matlab function. Download and usage instructions can be found in either of the following links:

- https://www.egichem.com/tools/calculators/d12-rg/
- https://github.com/EgiChem/D12_Rice_Gray

Table S1. Pure component data, compound name, chemical formula, CAS number, molecular weight, M, critical temperature, T_c, and volume, V_c.

Compound	Formula	CAS number	M (g mol^{-1})	T_c (K)	V_c (cm^3 mol^{-1})
[Bmim][BF4]	$C_8H_{15}N_2BF_4$	174501-65-6	226.02	643.20[a]	655.00[a]
[Bmim][bti]	$C_{10}H_{15}N_3F_6S_2O_4$	174899-83-3	419.4	1269.90[a]	990.10[a]
[Bmim][CF3SO3]	$C_9H_{15}N_2F_3SO_3$	174899-66-2	288.3	1023.50[a]	750.70[a]
[Bmim][Cl]	$C_8H_{15}N_2Cl$	79917-90-1	174.67	789.00[a]	568.80[a]
[Bmim][MeSO4]	$C_9H_{18}N_2SO_4$	401788-98-5	250.32	1081.60[a]	716.90[a]
[Bmim][OcSO4]	$C_{16}H_{32}N_2SO_4$	445473-58-5	348.5	1189.80[a]	1116.70[a]
[Bmim][PF6]	$C_8H_{15}N_2PF_6$	174501-64-5	284.18	719.40[a]	762.50[a]
[Emim][BF4]	$C_6H_{11}N_2BF_4$	143314-16-3	197.97	596.20[a]	540.80[a]
[Emim][bti]	$C_8H_{11}N_3F_6S_2O_4$	174899-82-2	391.31	1249.30[a]	875.90[a]
[Emim][C2H5SO4]	$C_8H_{16}N_2SO_4$	342573-75-5	236.3	1067.50[a]	659.80[a]
[Emim][C2N3]	$C_8H_{11}N_5$	370865-89-7	177.2	999.00[a]	597.80[a]
[Emim][CF3SO3]	$C_7H_{11}F_3N_2O_3S$	145022-44-2	260.2	992.30[a]	636.40[a]
[Emim][MDEGSO4]	$C_{11}H_{22}N_2SO_6$	790663-77-3	310.4	1162.90[a]	862.30[a]
[Hmim][bti]	$C_{12}H_{19}N_3F_6S_2O_4$	382150-50-7	447.42	1292.80[a]	1104.40[a]
[Omim][bti]	$C_{14}H_{23}N_3F_6S_2O_4$	178631-04-4	475.5	1317.80[a]	1218.60[a]
acetamide	C_2H_5NO	60-35-5	59.07	761.00[c]	215.00[c]
acetone	C_3H_6O	67-64-1	58.08	508.10[d]	209.00[d]
acetonitrile	C_2H_3N	75-05-8	41.05	545.50[d]	173.00[d]
acetylferrocene	$C_{12}H_{12}FeO$	1271–55–2	228.07	847.00[e]	569.20[f]
acridine	$C_{13}H_9N$	260-94-6	179.22	905.00[g]	543.00[g]
adamantanone	$C_{10}H_{14}O$	700-58-3	150.22	759.15[f]	368.22[f]
alanine	$C_3H_7NO_2$	56-41-7	89.09	698.48[h]	205.50[h]
alloisoleucine	$C_6H_{13}NO_2$	1509-34-8	131.17	762.89[h]	411.00[h]
allothreonine	$C_4H_9NO_3$	24830-94-2	119.12	827.59[h]	316.90[h]
allylbenzene	C_9H_{10}	300-57-2	118.18	639.86[i]	419.80[i]
aluminum acetylacetonate	$Al(C_5H_7O_2)_3$	13963-57-0	324.31	437.66[f]	881.69[f]
α-amino-*n*-butyric acid	$C_4H_9NO_2$	2835-81-6	103.12	719.32[h]	306.90[h]
ammonia	NH_3	7664-41-7	17.03	405.50[d]	72.50[d]
aniline	C_6H_7N	62-53-3	93.13	699.00[d]	274.00[d]
anisole	C_7H_8O	100-66-3	108.14	641.65[c]	337.00[c]
anthracene	$C_{14}H_{10}$	120-12-7	178.23	873.00[c]	554.00[c]

Compound	Formula	CAS number	M (g mol^{-1})	T_c (K)	V_c (cm^3 mol^{-1})
arabinose	$C_5H_{10}O_5$	147-81-9	150.13	1021.42[h]	370.95[h]
arachidonic acid (AA)	$C_{20}H_{32}O_2$	506-32-1	304.47	1013.42[h]	1093.20[h]
AA ethyl ester	$C_{22}H_{36}O_2$	1808-26-0	332.53	960.63[b]	1195.26[b]
argon	Ar	7440-37-1	39.95	150.80[d]	74.90[d]
astaxanthin	$C_{40}H_{52}O_4$	472-61-7	596.84	1148.51[j]	1877.50[j]
behenic acid ethyl ester	$C_{24}H_{48}O_2$	5908-87-2	368.64	984.94[b]	1394.66[b]
benzene	C_6H_6	71-43-2	78.11	562.20[d]	259.00[d]
benzoic acid	$C_7H_6O_2$	65-85-0	122.12	752.00[d]	341.00[d]
benzonitrile	C_7H_5N	100-47-0	103.12	699.35[c]	339.00[c]
benzothiophene	C_8H_6S	95-15-8	134.2	764.00[k]	379.00[k]
benzyl acetate	$C_9H_{10}O_2$	140-11-4	150.18	699.00[c]	449.00[c]
benzylacetone	$C_{10}H_{12}O$	2550-26-7	148.2	722.51[i]	500.50[i]
biphenyl	$C_{12}H_{10}$	92-52-4	154.21	789.00[d]	502.00[d]
2-bromoanisole	C_7H_7BrO	578-57-4	187.04	737.58[i]	378.05[i]
bromobenzene	C_6H_5Br	108-86-1	157.01	670.00[d]	324.00[d]
n-butane	C_4H_{10}	106-97-8	58.12	425.20[d]	255.00[d]
1,2-butanediol	$C_4H_{10}O_2$	584-03-2	90.12	622.14[c]	291.50[c]
1,3-butanediol	$C_4H_{10}O_2$	107-88-0	90.12	643.00[c]	292.00[c]
1,4-butanediol	$C_4H_{10}O_2$	111-63-4	90.12	667.00[c]	297.00[c]
1-butanol	$C_4H_{10}O$	71-36-3	74.12	563.10[d]	275.00[d]
2-methyl-1-butanol	$C_5H_{12}O$	137-32-6	88.15	565.00[c]	327.00[c]
2-methyl-2-butanol	$C_5H_{12}O$	75-85-4	88.15	545.15[c]	327.00[c]
3-methyl-1-butanol	$C_5H_{12}O$	123-51-3	88.15	579.45[c]	327.00[c]
3-methyl-2-butanol	$C_5H_{12}O$	598-75-4	88.15	574.00[c]	327.00[c]
n-butanol	$C_{410}O$	71-36-3	74.12	563.10[d]	275.00[d]
2-butanone	C_4H_8O	78-93-3	72.11	536.80[d]	267.00[d]
N-(4-methoxybenzylidene)-4-*n*-butylaniline	$C_{18}H_{21}NO$	26227-73-6	267.37	962.06[f]	592.93[f]
n-butylbenzene	$C_{10}H_{14}$	104-51-8	134.22	660.50[d]	497.00[d]
sec-butylbenzene	$C_{10}H_{14}$	135-98-8	134.22	672.06[l]	478.37[l]
tert-butylbenzene	$C_{10}H_{14}$	98-06-6	134.22	660.00[d]	492.00[c]
ethyl ester	$C_6H_{12}O_2$	105-54-4	116.2	579.00[m]	400.00[m]
caffeine	$C_8H_{10}N_4O_2$	58-08-2	194.2	855.60[m]	488.00[m]
camphor	Cl_0H_{160}	76-22-2	152.24	709.00[c]	460.00[c]
capric acid ethyl ester	$C_{12}H_{24}O_2$	110-38-3	200	699.30[m]	733.50[m]
caprylic acid ethyl ester	$C_{10}H_{20}O_2$	106-32-1	172.3	655.70[m]	621.50[m]
carbon dioxide	CO_2	124-38-9	44.01	304.10[d]	93.90[d]
carbon disulfide	CS_2	75-15-0	76.13	552.00[d]	160.00[d]
carbon monoxide	CO	630-08-0	28.01	132.90[d]	93.20[d]
carbon tetrabromide	CBr_4	558-13-4	331.63	724.91[c]	328.50[c]

Compound	Formula	CAS number	M (g mol^{-1})	T_c (K)	V_c (cm^3 mol^{-1})
carbon tetrachloride	CCl_4	56-23-5	153.82	556.40[d]	275.90[d]
β-carotene	$C_{40}H_{56}$	7235-40-7	536.88	1450.76[h]	1934.95[h]
L-carvone	$C_{10}H_{14}O$	6485-40-1	150.22	709.40[n]	504.65[n]
chlorobenzene	C_6H_5Cl	108-90-7	112.56	632.40[d]	308.00[d]
p-chloronitrobenzene	$C_6H_4ClNO_2$	100-00-5	157.56	751.00[c]	432.00[c]
chlorotrifluoromethane	$CClF_3$	75-72-9	104.46	302.00[d]	180.40[d]
chromium(III) acetylacetonate	$Cr(acac)_3$	21679-31-2	349.32	858.85[f]	627.04[f]
chrysene	$C_{18}H_{12}$	218-01-9	228.29	979.00[c]	690.00[c]
cinnamic acid	$C_9H_8O_2$	140-10-3	148.161	797.00[g]	446.00[g]
citral	$C_{10}H_{16}O$	5392-40-5	152.24	692.70[h]	591.00[h]
cobalt(III) acetylacetonate	$C_{15}H_{21}CoO_6$	21679-46-9	356.26	573.48[f]	640.95[f]
copper(II) trifluoroacetylacetonate	$C_{10}H_8CuF_6O_4$	14324-82-4	369.7	412.85[f]	441.13[f]
12-crown-4	$C_8H_{16}O_4$	294-93-9	176.21	780.66[h]	444.75[h]
15-crown-5	$C_{10}H_{20}O_5$	33100-27-5	220.27	876.80[h]	548.75[h]
18-crown-6	$C_{12}H_{24}O_6$	17455-13-9	264.32	970.51[h]	652.75[h]
dicyclohexano-18-crown-6	$C_{20}H_{36}O_6$	16069-36-6	372.5	1177.47[h]	1002.75[h]
dicyclohexano-24-crown-8	$C_{24}H_{44}O_8$	17455-23-1	460.61	1357.66[h]	1210.75[h]
dibenzo-24-crown-8	$C_{24}H_{32}O_8$	14174-09-5	448.51	1396.77[h]	1174.35[h]
α-cyclodextrin	$C_{36}H_{60}O_{30}$	10016-20-3	972.84	2580.29[f]	1794.01[f]
β-cyclodextrin	$C_{42}H_{70}O_{35}$	7585-39-9	1134.98	2790.00[f]	2089.68[f]
cycloheptanone	$C_7H_{12}O$	502-42-1	112.17	671.19[f]	297.87[f]
cyclohexane	C_6H_{12}	110-82-7	84.16	553.50[d]	308.00[d]
cyclononanone	$C_9H_{16}O$	3350-30-9	140.22	702.10[f]	380.74[f]
cyclopentane	C_5H_{10}	287-92-3	70.14	511.70[d]	260.00[d]
cyclopentanone	C_5H_8O	120-92-3	84.12	626.00[c]	258.00[c]
n-decane	$C_{10}H_{22}$	124-18-5	142.29	617.70[d]	603.00[d]
deuterium oxide	D_2O	7789-20-0	20.03	643.89[d]	56.26[d]
dibenzothiophene	$C_{12}H_8S$	132-65-0	184.26	897.00[k]	512.00[k]
dibenzyl ether	$C_{14}H_{14}O$	103-50-4	198.26	777.00[c]	608.00[c]
1,3-dibromobenzene	$C_6H_4Br_2$	108-36-1	235.91	761.00[c]	372.00[c]
1,2-dichlorobenzene	$C_6H_4Cl_2$	95-50-1	147	729.00[d]	360.00[d]
1,3-dichlorobenzene	$C_6H_4Cl_2$	541-73-1	147	683.95[c]	351.00[c]
p-dichlorobenzene	$C_6H_4Cl_2$	106-46-7	147	684.75[c]	351.00[c]
diethanolamine	$C_4H_{11}NO_2$	111-42-2	105.14	736.60[k]	349.00[k]
diethyl ether	$C_4H_{10}O$	60-29-7	74.12	466.70[d]	280.00[d]
1,2-diethylbenzene	$C_{10}H_{14}$	135-01-3	134.22	668.00[c]	502.00[c]
1,4-diethylbenzene	$C_{10}H_{14}$	105-05-5	134.22	657.96[c]	497.00[c]
diethylene glycol	$C_4H_{10}O_3$	111-46-6	106.12	744.60[c]	312.00[c]
N,*N*-diethylethanolamine	$C_6H_{15}NO$	100-37-8	117.19	592.00[g]	401.00[g]

Compound	Formula	CAS number	M (g mol^{-1})	T_c (K)	V_c (cm^3 mol^{-1})
o-difluorobenzene	$C_6H_4F_2$	367-11-3	114.09	554.46[c]	299.50[c]
p-difluorobenzene	$C_6H_4F_2$	540-36-3	114.09	556.00[c]	299.50[c]
diglycolamine	$C_4H_{11}NO_2$	929-06-6	105.14	699.00[c]	330.00[c]
diisopropanolamine	$C_6H_{15}NO_2$	110-97-4	133.19	672.00[k]	454.00[k]
diisopropyl ether	$C_6H_{14}O$	108-20-3	102.18	500.30[d]	386.00[d]
2,4-dimethyl-3-pentanone	$C_7H_{14}O$	565-80-0	114.19	576.00[g]	416.00[g]
2,3-dimethylaniline	$C_8H_{11}N$	87-59-2	121.18	717.00[i]	400.38[i]
2,6-dimethylaniline	$C_8H_{11}N$	87-62-7	121.18	722.00[o]	400.38[i]
9,10-dimethylanthracene	$C_{16}H_{14}$	781-43-1	206.29	899.22[h]	724.55[h]
2,3-dimethylbutane	C_6H_{14}	79-29-8	86.18	500.00[d]	358.00[d]
1,1′-dimethylferrocene	$C_{12}H_{14}Fe$	1291-47-0	214.09	514.45[f]	400.64[f]
2,3-dimethylnaphthalene	$C_{12}H_{12}$	581-40-8	156.23	777.78[c]	521.50[c]
2,6-dimethylnaphthalene	$C_{12}H_{12}$	581-42-0	156.23	777.00[c]	520.00[c]
2,7-dimethylnaphthalene	$C_{12}H_{12}$	582-16-1	156.23	778.00[c]	520.00[c]
2,4-dimethylphenol	$C_8H_{10}O$	105-67-9	122.17	707.60[d]	390.00[c]
diolein	$C_{39}H_{72}O_5$	2465-32-9	621.99	1025.00[g]	2150.00[g]
dipropylene glycol	$C_6H_{14}O_3$	25265-71-8	134.18	654.00[c]	415.00[c]
disperse blue 14	$C_{16}H_{14}N_2O_2$	2475-44-7	266	1137.33[j]	765.50[j]
disperse orange 11	$C_{15}H_{11}NO_2$	82-28-0	237.25	1103.62[j]	670.00[j]
1,3-divinylbenzene	$C_{10}H_{10}$	108-57-6	130.19	692.00[c]	440.00[c]
dimethylethanolamine (DMEA)	$C_4H_{11}NO$	108-01-0	89.14	571.82[c]	300.00[c]
docosahexaenoic acid (DHA)	$C_{22}H_{32}O_2$	6217-54-5	328.49	833.67[h]	1164.30[h]
DHA ethyl ester	$C_{24}H_{36}O_2$	84494-72-4	356.55	828.45[h]	1276.40[h]
DHA methyl ester	$C_{23}H_{34}O_2$	2566-90-7	342.52	844.78[h]	1220.85[h]
n-dodecane	$C_{12}H_{26}$	112-40-3	170.34	658.20[d]	713.00[d]
n-eicosane	$C_{20}H_{42}$	112-95-8	282.56	767.00[d]	1190.00[c]
eicosapentaenoic acid (EPA)	$C_{20}H_{30}O_2$	10417-94-4	302.46	1020.90[b]	1059.15[b]
EPA ethyl ester	$C_{22}H_{34}O_2$	84494-70-2	330.51	968.16[b]	1173.16[b]
EPA methyl ester	$C_{21}H_{32}O_2$	2734-47-6	316.48	890.55[b]	1187.03[b]
meso-erythritol	$C_4H_{10}O_4$	149-32-6	122.12	940.29[h]	313.95[h]
ethane	C_2H_6	74-84-0	30.07	305.40[d]	148.30[d]
ethanol	C_2H_6O	64-17-5	46.07	513.90[d]	167.10[d]
ethyl acetate	$C_4H_8O_2$	141-78-6	88.11	523.30[g]	286.00[g]
ethyl benzoate	$C_9H_{10}O_2$	93-89-0	150.18	668.70[d]	489.00[c]
ethylbenzene	C_8H_{10}	100-41-4	106.17	617.20[d]	374.00[d]
ethylene	C_2H_4	74-85-1	28.05	282.40[d]	130.40[d]
ethylene glycol	$C_2H_6O_2$	107-21-1	62.07	645.00[c]	191.00[c]
ethylferrocene	$C_{12}H_{14}Fe$	1273-89-8	214.08	554.21[f]	400.64[f]
1-ethylnaphthalene	$C_{12}H_{12}$	1127-76-0	156.23	776.00[c]	520.00[c]

Compound	Formula	CAS number	M (g mol-1)	T_c (K)	V_c (cm3 mol-1)
2-ethyltoluene	C_9H_{12}	611-14-3	120.19	651.00[d]	460.00[d]
3-ethyltoluene	C_9H_{12}	620-14-4	120.19	637.00[d]	490.00[d]
4-ethyltoluene	C_9H_{12}	622-96-8	120.19	640.00[d]	470.00[d]
eucalyptol	$C_{10}H_{18}O$	470-82-6	154.25	695.50[p].	509.50[p].
eugenol	$C_{10}H_{12}O_2$	97-53-0	164.2	735.31[i]	447.23[i]
ferrocene	$C_{10}H_{10}Fe$	102-54-5	186.04	786.27[f]	317.77[f]
2-fluoroanisole	C_7H_7FO	321-28-8	126.13	644.81[i]	328.87[i]
fluorobenzene	C_6H_5F	462-06-6	96.1	560.10[d]	269.00[d]
3-fluorophenol	C_6H_5FO	372-20-3	112.1	665.54[h]	339.60[h]
formamide	CH_3NO	75-12-7	45.04	771.00[c]	163.00[c]
fructose	$C_6H_{12}O_6$	57-48-7	180.16	1242.06[f]	357.45[f]
5-(hydroxymethyl) furfural	$C_6H_6O_3$	67-47-0	126.11	737.30[k]	343.00[k]
furfural	$C_5H_4O_2$	98-01-1	96.08	670.15[k]	252.00[k]
galactose	$C_6H_{12}O_6$	59-23-4	180.16	1059.54[k]	459.00[k]
gallic acid	$C_7H_6O_5$	149-91-7	170.12	1136.70[q]	276.20[q]
geraniol	$C_{10}H_{18}O$	106-24-1	154.25	688.44[h]	571.30[h]
glucose	C_6H_{1206}	50-99-7	180.16	755.00[g]	414.00[g]
glycerol	$C_3H_8O_3$	56-81-5	92.09	723.00[c]	264.00[c]
glycine	$C_2H_5NO_2$	56-40-6	75.07	1021.00[r]	234.00[r]
guaiacol	$C_7H_8O_2$	90-05-1	124.14	697.00[k]	353.00[k]
n-heptane	C_7H_{16}	142-82-5	100.2	540.30[d]	432.00[d]
2-heptanone	$C_7H_{14}O$	110-43-0	114.19	611.50[d]	421.00[c]
4-heptanone	$C_7H_{14}O$	123-19-3	114.19	595.31[c]	433.50[c]
hexachlorobenzene	C_6Cl_6	118-74-1	284.78	825.00[c]	526.00[c]
n-hexadecane	$C_{16}H_{34}$	544-76-3	226.45	722.00[d]	930.00[d]
1-hexadecene	$C_{16}H_{32}$	629-73-2	224.43	722.00[k]	933.00[k]
1,1,1,5,5,5-hexafluoroacetylacetone	$C_5H_2F_6O_2$	1552-22-1	208.06	569.07[b]	406.05[b]
hexafluorobenzene	C_6F_6	392-56-3	186.06	516.70[d]	335.00[d]
n-hexane	C_6H_{14}	110-54-3	86.18	507.50[d]	370.00[d]
1,2,6-hexanetriol	$C_6H_{14}O_3$	106-69-4	134.18	844.91[h]	415.05[h]
homoserine	$C_4H_9NO_3$	672-15-1	119.12	823.94[h]	323.90[h]
hydrogen	H_2	1333-74-0	2.02	33.00[d]	64.30[d]
hydrogen sulfide	H_2S	04/06/7783	34.08	373.20[d]	98.60[d]
Ibuprofen	$C_{13}H_{18}O_2$	15687-27-1	206.29	769.63[h]	686.35[h]
indole	C_8H_7N	204-420-7	117.15	790.00[t]	431.00[t]
myo-inositol	$C_6H_{12}O_6$	87-89-8	180.16	850.00[g]	412.00[g]
iodobenzene	C_6H_5I	591-50-4	204.01	721.00[d]	351.00[d]
isobutylbenzene	$C_{10}H_{14}$	538-93-2	134.22	650.00[l]	480.00[l]
isobutyramide	C_4H_9NO	127-19-5	87.12	658.00[g]	321.00[g]

Compound	Formula	CAS number	M (g mol^{-1})	T_c (K)	V_c (cm^3 mol^{-1})
isoleucine	$C_6H_{13}NO_2$	73-32-5	131.17	761.52[h]	412.50[h]
krypton	Kr	7439-90-9	83.8	209.40[d]	91.20[d]
lactose	$C_{12}H_{22}O_{11}$	63-42-3	342.3	1431.89[f]	653.12[f]
leucine	$C_6H_{13}O_2N$	61-90-5	131.17	761.52[h]	412.50[h]
tert-leucine	$C_6H_{13}NO_2$	20859-02-3	131.17	766.89[h]	404.00[h]
D-limonene	$C_{10}H_{16}$	5989-27-5	136.24	660.00[c]	524.00[c]
linalool	$C_{10}H_{18}O$	78-70-6	154.25	645.80[h]	558.00[h]
linoleic acid	$C_{18}H_{32}O_2$	60-33-3	280.45	775.00[g]	990.00[g]
linoleic acid methyl ester	$C_{19}H_{34}O_2$	112-63-0	294.48	870.78[b]	1070.95[b]
α-Linolenic acid	$C_{18}H_{30}O_2$	463-40-1	278.44	780.00[g]	1070.00[g]
γ-linolenic acid	$C_{18}H_{30}O_2$	506-26-3	278.44	958.98[b]	992.35[b]
γ-linolenic acid ethyl ester	$C_{20}H_{34}O_2$	31450-14-3	306.48	937.01[f]	797.37[f]
γ-linolenic acid methyl ester	$C_{19}H_{32}O_2$	16326-32-2	292.46	882.79[b]	1050.86[b]
mannitol	$C_6H_{14}O_6$	69-65-8	182.17	1246.06[h]	445.05[h]
mannose	$C_6H_{12}O_6$	3458-28-4	180.16	1059.54[k]	459.00[k]
monoethanolamine (MEA)	C_2H_7NO	141-43-5	61.08	638.00[c]	225.00[c]
L-menthone	$C_{10}H_{18}O$	14073-97-3	154.25	699.44[n]	525.24[n]
methane	CH_4	74-82-8	16.04	190.40[d]	99.20[d]
methanol	CH_4O	67-56-1	32.04	512.60[d]	118.00[d]
methyl bromide	CH_3Br	74-83-9	94.94	467.00[c]	156.00[c]
methyl chloride	CH_3Cl	74-87-3	50.49	416.30[d]	138.90[d]
methyl fluoride	CH_3F	593-53-3	34.03	315.00[d]	113.20[d]
2-methylanisole	$C_8H_{10}O$	578-58-5	122.17	648.79[i]	371.70[i]
4-methylanisole	$C_8H_{10}O$	104-93-8	122.17	655.36[i]	371.70[i]
3-methylbutylbenzene	$C_{11}H_{15}$	2049-94-7	148.25	672.06[l]	542.47[l]
methylcyclopentane	C_6H_{12}	96-37-7	84.16	532.70[d]	319.00[d]
n-methyldiethanolamine	$C_5H_{13}NO_2$	105-59-9	119.16	675.00[k]	368.00[k]
1-methylnaphthalene	$C_{11}H_{10}$	90-12-0	142.2	772.00[k]	465.00[k]
N-methylpyrrolidone	C_5H_9NO	872-50-4	99.13	724.00[c]	316.00[c]
monoisopropanolamine (MIPA)	C_3H_9NO	78-96-6	75.11	614.00[c]	278.00[c]
monoolein	$C_{21}H_{40}O_4$	111-03-5	356.55	885.00[g]	1210.00[g]
myristic acid ethyl ester	$C_{16}H_{32}O_2$	124-06-1	256.43	789.35[b]	950.66[b]
myristoleic acid	$C_{14}H_{26}O_2$	544-64-9	226.36	854.23[h]	819.90[h]
myristoleic acid methyl ester	$C_{15}H_{28}O_2$	56219-06-8	240.39	777.79[h]	876.45[h]
naphthalene	$C_{10}H_8$	91-20-3	128.17	748.40[d]	413.00[d]
1-naphthol	$C_{10}H_8O$	90-15-3	144.17	802.00[c]	375.50[c]
2-naphthol	$C_{10}H_8O$	135-19-3	144.17	811.40[m]	375.50[m]
neon	Ne	09/01/7440	20.18	44.40[d]	41.60[d]
nickel(II) acetylacetonate	$Ni(acac)_2$	3264-82-2	256.91	654.02[f]	431.20[f]

Compound	Formula	CAS number	M (g mol-1)	T_c (K)	V_c (cm3 mol-1)
2-nitroanisole	$C_7H_7NO_3$	91-23-6	153.14	782.00[c]	422.00[c]
nitrobenzene	$C_6H_5NO_2$	98-95-3	123.11	719.00[c]	349.00[c]
nitrogen	N_2	7727-37-9	28.01	126.20[d]	89.80[d]
3-nitrotoluene	$C_7H_7NO_2$	99-08-1	137.14	734.00[c]	441.00[c]
nitrous oxide	N_2O	10024-97-2	44.01	309.60[d]	97.40[d]
n-nonane	C_9H_{20}	111-84-2	128.26	594.60[d]	548.00[d]
2-nonanone	$C_9H_{18}O$	821-55-6	142.24	644.29[c]	545.50[c]
5-nonanone	$C_9H_{18}O$	502-56-7	142.24	640.00[g]	560.00[g]
norleucine	$C_6H_{13}NO_2$	327-57-1	131.17	757.63[h]	418.50[h]
norvaline	$C_5H_{11}NO_2$	09/12/2013	117.15	737.84[h]	362.50[h]
octafluorotoluene	C_7F_8	434-64-0	236.06	534.47[o]	428.00[o]
n-octane	C_8H_{18}	111-65-9	114.23	568.80[d]	492.00[d]
1-octene	C_8H_{16}	111-66-0	112.22	566.70[d]	464.00[d]
oleic acid	$C_{18}H_{34}O_2$	112-80-1	282.47	781.00[g]	1000.00[g]
oleic acid ethyl ester	$C_{20}H_{38}O_2$	111-62-6	310.52	891.97[h]	1154.20[h]
oleic acid methyl ester	$C_{19}H_{36}O_2$	112-62-9	296.49	868.65[h]	1098.65[h]
oxygen	O_2	7782-44-7	32	154.60[d]	73.40[d]
palladium(II) acetylacetonate	$C_{10}H_{14}O_4Pd$	14024-61-4	304.64	651.12[f]	435.41[f]
palmitic acid ethyl ester	$C_{18}H_{36}O_2$	628-97-7	284.48	835.62[b]	1061.66[b]
pentaerythritol	C_5H_{1204}	115-77-5	136.15	780.00[c]	381.00[c]
pentafluorobenzene	C_6HF_5	363-72-4	168.07	530.97[o]	324.00[o]
n-pentane	C_5H_{12}	109-66-0	72.15	469.70[d]	304.00[d]
2-methyl-2,4-pentanediol	$C_6H_{14}O_2$	107-41-5	118.18	621.00[g]	398.00[g]
1-pentanol	$C_5H_{12}O$	71-41-0	88.15	588.20[d]	326.00[d]
2-pentanol	$C_5H_{12}O$	6032-29-7	88.15	552.00[c]	327.00[c]
3-pentanol	$C_5H_{12}O$	584-02-1	88.15	547.00[c]	327.00[c]
2-pentanone	$C_5H_{10}O$	107-87-9	86.13	561.10[d]	301.00[d]
3-pentanone	$C_5H_{10}O$	96-22-0	86.13	561.00[d]	336.00[d]
n-pentylbenzene	$C_{11}H_{16}$	538-68-1	148.25	679.90[c]	550.00[c]
phenanthrene	$C_{14}H_{10}$	85-01-8	178.23	873.00[d]	554.00[d]
phenol	C_6H_6O	108-95-2	94.11	694.20[d]	229.00[d]
phenylacetic acid	$C_8H_8O_2$	103-82-2	136.15	783.55[h]	422.60[h]
phenylacetylene	C_8H_6	536-74-3	102.14	655.43[c]	337.50[c]
phenylbutazone	$C_{19}H_{20}N_2O_2$	50-33-9	308.38	861.18[h]	933.55[h]
1-phenyldodecane	$C_{18}H_{30}$	123-01-3	246.44	774.26[c]	1000.00[c]
1-phenylethanol	$C_8H_{10}O$	98-85-1	122.17	675.30[i]	392.15[j]
2-phenylethanol	$C_8H_{10}O$	60-12-8	122.17	684.00[c]	387.00[c]
2-phenylethyl acetate	$C_{10}H_{12}O_2$	103-45-7	164.1	712.23[i]	524.15[i]
1-phenylhexane	$C_{12}H_{18}$	1077-16-3	162.28	698.00[c]	618.00[c]

Compound	Formula	CAS number	M (g mol-1)	T_c (K)	V_c (cm3 mol-1)
phenylmethanol	C_7H_8O	100-51-6	108.14	720.20[d]	335.00[c]
1-phenyloctane	$C_{14}H_{22}$	2189-60-8	190.33	729.00[c]	703.00[c]
3-phenylpropyl acetate	$C_{11}H_{14}O_2$	122-72-5	178.3	718.70[i]	580.37[i]
α-pinene	$C_{10}H_{16}$	80-56-8	136.24	632.00[c]	504.00[c]
β-pinene	$C_{10}H_{16}$	127-91-3	136.24	643.00[c]	506.00[c]
2-piperidineethanol	$C_7H_{15}NO$	1484-84-0	129.2	745.14[h]	418.40[h]
potassium chloride	KCl	7447-40-7	74.55	3470.00[g]	625.00[g]
propanamide	C_3H_7NO	79-16-3	73.09	718.00[c]	267.00[c]
propane	C_3H_8	74-98-6	44.09	369.80[d]	203.00[d]
1-propanol	C_3H_8O	71-23-8	60.1	536.80[d]	219.00[d]
2-phenyl-1-propanol	$C_9H_{12}O$	1123-85-9	136.2	662.02[i]	443.23[i]
3-phenyl-1-propanol	$C_9H_{12}O$	122-97-4	136.2	702.30[i]	455.45[i]
2,2-dimethyl-1-propanol	$C_5H_{12}O$	75-84-3	88.15	550.00[c]	327.00[c]
2-amino-2-methyl-1-propanol	$C_4H_{11}NO$	124-68-5	89.14	619.80[k]	315.00[k]
2-methyl-2-propanol	$C_4H_{10}O$	75-65-0	74.12	506.20[c]	275.00[c]
2-propanol	C_3H_8O	67-63-0	60.1	508.30[d]	220.00[d]
propene	C_3H_6	115-07-1	42.08	364.90[d]	181.00[d]
i-propylbenzene	C_9H_{12}	98-82-8	120.19	631.10[d]	427.70[c]
n-propylbenzene	C_9H_{12}	103-65-1	120.19	638.20[d]	440.00[d]
propylene glycol	$C_3H_8O_2$	57-55-6	76.09	626.00[c]	239.00[c]
pyrene	$C_{16}H_{10}$	129-00-0	202.25	936.00[c]	630.00[c]
piperazine (PZ)	$C_4H_{10}N_2$	110-85-0	86.14	638.00[c]	310.00[c]
quercetin	$C_{15}H_{10}O_7$	117-39-5	302.24	1468.74[j]	730.50[j]
radon	Rn	10043-92-2	222	377.40[c]	140.00[c]
salicylic acid	C_7H_{603}	69-72-7	138.12	739.00[c]	364.00[c]
serine	$C_3H_7NO_3$	56-45-1	105.09	804.97[h]	268.35[h]
squalene	$C_{30}H_{50}$	111-02-4	410.73	716.50[u]	1601.00[j]
stearic acid	$C_{18}H_{36}O_2$	57-11-4	284.48	803.00[k]	1140.00[k]
stearic acid ethyl ester	$C_{20}H_{40}O_2$	111-61-5	312.53	777.90[k]	1380.00[k]
styrene	C_8H_8	100-42-5	104.15	647.00[d]	352.00[c]
sucrose	$C_{12}H_{22011}$	57-50-1	342.3	1086.00[g]	761.00[g]
sulfolane	$C_4H_8O_2S$	126-33-0	120.17	849.00[c]	300.00[c]
sulfur dioxide	SO_2	05/09/7446	64.06	430.75[c]	122.00[c]
sulfur hexafluoride	SF_6	2551-62-4	146.05	318.70[d]	198.80[d]
tetrabutyltin	$C_{16}H_{36}Sn$	1461-25-2	347.17	767.97[f]	760.75[f]
n-tetradecane	$C_{14}H_{30}$	629-59-4	198.39	693.00[d]	830.00[d]
1-tetradecene	$C_{14}H_{28}$	1120-36-1	196.37	691.00[k]	865.00[k]
tetraethylene glycol	$C_8H_{18}O_5$	112-60-7	194.23	722.00[c]	564.00[c]
tetraethyltin	$C_8H_{20}Sn$	597-64-8	234.95	655.92[f]	429.28[f]

Compound	Formula	CAS number	M (g mol-1)	T_c (K)	V_c (cm3 mol-1)
1,2,3,5-tetrafluorobenzene	$C_6H_2F_4$	2367-82-0	150.08	555.49[h]	351.05[h]
1,2,4,5-tetrafluorobenzene	$C_6H_2F_4$	327-54-8	150.07	535.25[o]	351.05[h]
tetrahydrofuran	C_4H_8O	109-99-9	72.11	540.10[d]	224.00[d]
2,2,4,4-tetramethyl-3-pentanone	$C_9H_{18}O$	815-24-7	142.24	627.18[f]	407.72[f]
tetramethyltin	$C_4H_{12}Sn$	594-27-4	178.85	511.77[f]	263.54[f]
tetrapropyltin	$C_{12}H_{28}Sn$	2176-98-9	291.06	759.88[f]	595.01[f]
thenoyltrifluoroacetone	$C_8H_5F_3O_2S$	326-91-0	222.18	838.69[f]	428.15[f]
threonine	$C_4H_9NO_3$	72-19-5	119.12	859.80[h]	304.40[h]
α-tocopherol	$C_{29}H_{50}O_2$	10191-41-0	430.71	915.76[r]	1534.20[r]
toluene	C_7H_8	108-88-3	92.14	591.80[d]	316.00[d]
triarachidonin	$C_{63}H_{98}O_6$	23314-57-0	951.45	1499.66[f]	2341.53[f]
1,2,4-trichlorobenzene	$C_6H_3Cl_3$	120-82-1	181.45	725.00[c]	395.00[c]
trierucin	$C_{69}H_{128}O_6$	2752-99-0	1053.75	1549.28[f]	2832.93[f]
triethanolamine	$C_6H_{15}N_{03}$	102-71-6	149.19	772.10[g]	472.00[g]
triethylene glycol	$C_6H_{14}O_4$	112-27-6	150.18	700.00[c]	443.00[c]
trifluoroacetylacetone	$C_5H_5F_3O_2$	367-57-7	154.09	594.02[b]	365.58[b]
1,2,4-trifluorobenzene	$C_6H_3F_3$	367-23-7	132.09	558.22[h]	335.05[h]
1,3,5-trimethylbenzene	C_9H_{12}	108-67-8	120.19	637.30[d]	433.00[c]
2,2,4-trimethylpentane	C_8H_{18}	540-84-1	144.23	543.80[c]	468.00[c]
trinervonin	$C_{75}H_{140}O_6$	81913-24-8	1137.91	1601.10[f]	3081.54[f]
triolein	$C_{57}H_{104}O_6$	122-32-7	885.43	1640.00[g]	3090.00[g]
s-trioxane	$C_3H_6O_3$	110-88-3	90.08	604.00[c]	206.00[c]
ubiquinone CoQ10	$C_{59}H_{90}O_4$	303-98-0	863.34	1522.50[f]	2146.17[f]
n-undecane	$C_{11}H_{24}$	1120-21-4	156.31	638.80[d]	660.00[d]
6-undecanone	$C_{11}H_{22}O$	927-49-1	170.3	678.01[g]	657.50[g]
valine	$C_5H_{11}NO_2$	72-18-4	117.15	741.96[h]	356.50[h]
vanadyl(II) acetylacetonate	$VO(acac)_2$	3153-26-2	265.16	652.70[f]	445.30[f]
vanillin	$C_8H_8O_3$	121-33-5	152.15	777.00[c]	415.00[c]
vitamin K_1	$C_{31}H_{48}O_2$	84-80-0	452.71	1329.54[h]	1620.20[h]
vitamin K_3	$C_{11}H_8O_2$	58-27-5	172.18	893.85[h]	537.20[h]
water	H_2O	7732-18-5	18.02	647.30[d]	57.10[d]
xenon	Xe	7440-63-3	131.3	289.70[d]	118.40[d]
5-*tert*-butyl-*m*-xylene	$C_{12}H_{18}$	98-19-1	162.28	684.85[i]	591.75[i]
m-xylene	C_8H_{10}	108-38-3	106.17	617.10[d]	376.00[d]
o-xylene	C_8H_{10}	95-47-6	106.17	630.30[d]	369.00[d]
p-xylene	C_8H_{10}	106-42-3	106.17	616.20[d]	379.00[d]
xylitol	$C_5H_{12}O_5$	87-99-0	152.15	1032.99[k]	399.00[k]
xylose	$C_5H_{10}O_5$	25990-60-7	150.13	953.50[k]	388.00[k]

[a]Taken from Valderrama and Rojas [1]; [b]Average of the values by the Joback [2–4]and Somayajulu [5] methods; [c]Taken from Yaws (1998) [6]; [d]Taken from Reid *et al.* [7]; [e]Taken from Nikitin and Popov [8];

[f]Estimated by the Klincewicz method [9,10]; [g]Taken from Yaws (2008) [6]; [h]Average of the values by the Joback [2–4] and Ambrose [9,11] methods; [i]Average of the values by the Joback [2–4] and Wen-Qiang [12] methods; [j]Estimated by the Joback method [2–4]; [l]Taken from Pizarro et al [13]; [k]Taken from DIPPR database [14]; [m]Taken from Table 4 of Liu and Ruckenstein [15]; [n]Average of the values by the Joback [2–4] and Constantinou-Gani [16] methods; [o]Taken from Korea Thermophysical Properties Data Bank (KDB) [17]; [p]Taken from Zêzere *et al.* [18]; [q]Taken from Leite *et al.* [19]; [r]Taken from Aspen database; [s]Taken from Poling [4]; [t]Taken from Magalhães *et al.* [20]; [u]Taken from Catchpole *et al.* [21].

Table S2. Polar solvents systems studied, number of data point (NDP), reduced number density range ($\rho^*_{n,1}$ range), absolute temperature range (T range), binary interaction parameters (k_{12} and B_{12}), average absolute relative deviations (AARD) and source of the diffusion data.

Solvent	Solute	NDP	$\rho^*_{n,1}$ range	T range (K)	k_{12}	B_{12}	AARD (%)	Data source
**Polar systems**								
acetone	benzene	4	0.817-0.918	268.20-328.20	0.25605	2.30400	0.35	[22]
	biphenyl	7	0.817-0.918	268.20-328.20	0.18832	1.21603	0.4	[22]
	chlorobenzene	12	0.817-0.918	268.20-328.20	0.25829	2.14041	0.67	[22]
	ethylbenzene	7	0.817-0.918	268.20-328.20	0.26830	2.25498	0.46	[22]
	naphthalene	5	0.817-0.918	268.20-328.20	0.22500	1.50436	0.46	[22]
	n-propylbenzene	5	0.817-0.918	268.20-328.20	0.23700	1.78685	0.74	[22]
	toluene	5	0.817-0.918	268.20-328.20	0.27635	2.43819	0.4	[22]
	1,2,4-trichlorobenzene	6	0.817-0.918	268.20-328.20	0.21125	1.63602	0.93	[22]
	1,3,5-trimethylbenzene	5	0.817-0.918	268.20-328.20	0.24201	1.96628	0.46	[22]
	water	6	0.817-0.867	298.15-328.15	0.62778	34.61652	0.89	[23]
n-butanol	ammonia	6	0.866-0.938	298.15-348.15	-3.91416	-4.37610	8.4	[24]
	carbon dioxide	12	0.858-0.938	298.15-348.15	-0.57025	-1.67764	4.32	[24]
	propane	8	0.861-0.940	298.15-348.15	-0.90270	-2.76134	3.09	[24]
	propene	7	0.859-0.943	298.15-348.20	-0.86623	-2.83098	3.29	[24]
chlorobenzene	propene	7	0.886-0.956	298.15-348.20	0.16735	3.75283	1.2	[25,26]
chlorotrifluoromethane	acetone	7	0.191-0.464	313.15-313.15	0.61264	13.85654	2.31	[27]
	1,3-dibromobenzene	6	0.191-0.485	318.15-318.15	0.50307	6.47803	5.96	[27]
	p-xylene	12	0.191-0.485	318.15-318.15	0.81469	58.54792	3	[27]
deuterium oxide	oxygen	12	0.896-0.992	276.85-369.75	-0.21381	-5.54592	4.43	[28]

Solvent	Solute	NDP	$\rho_{n,1}^{*}$ range	T range (K)	k_{12}	B_{12}	AARD (%)	Data source
ethanol	acetylferrocene	7	0.830-0.843	322.90-332.50	-1.19730	-0.68531	0	[29]
	aluminum acetylacetonate	4	0.828-0.899	300.15-333.20	-1.17095	-1.60137	8.57	[30]
	ammonia	8	0.839-0.884	298.15-323.15	-2.19069	-2.94112	5.18	[24]
	benzene	3	0.406-0.906	278.20-554.00	-0.68969	-1.22218	8.73	[31,32]
	benzonitrile	3	0.841-0.868	303.00-323.00	-1.15661	-1.26926	1.69	[33]
	benzyl acetate	4	0.839-0.881	313.16-333.16	-0.91862	-0.88023	2.95	[34]
	1,2-butanediol	4	0.769-0.906	278.20-372.00	-2.25374	-1.76681	1.77	[32]
	1,4-butanediol	4	0.769-0.906	278.20-372.00	-2.42504	-1.64303	1.1	[32]
	1-butanol	4	0.769-0.906	278.20-372.00	-1.79960	-1.74126	1.09	[32]
	carbon dioxide	4	0.800-0.886	298.15-348.15	-0.24809	-0.92307	4.36	[24]
	chromium(III) acetylacetonate	3	0.827-0.875	299.15-333.15	-2.54330	-1.28065	1.12	[35,36]
	dibenzyl ether	4	0.839-0.881	313.16-333.16	-1.05058	-1.06518	3.68	[34]
	disperse blue 14	4	0.849-0.862	308.20-318.20	-2.53415	-1.23491	4.84	[37]
	disperse orange 11	4	0.836-0.862	308.20-328.20	-2.30560	-1.21107	0.47	[37]
	ethylene glycol	4	0.769-0.906	278.20-372.00	-2.33394	-1.70534	2.89	[32]
	eucalyptol	4	0.830-0.878	303.15-333.15	-1.29243	-1.20283	2.47	[18]
	gallic acid	8	0.830-0.878	303.15-333.15	-2.87189	-0.94638	3.66	[19]
	glycerol	11	0.769-0.906	278.20-372.00	-2.53053	-1.57400	1.1	[32]
	ibuprofen	9	0.840-0.890	298.15-333.15	-1.17781	-0.64238	6.75	[38]
	naphthalene	11	0.406-0.774	373.20-554.00	-0.33041	-0.57019	6.6	[31]
	nickel(II) acetylacetonate	10	0.827-0.865	307.15-333.15	-2.12763	-1.38225	1.45	[30]

Solvent	Solute	NDP	$\rho^*_{n,1}$ range	T range (K)	k_{12}	B_{12}	AARD (%)	Data source
	nitrous oxide	5	0.827-0.878	298.10-333.00	-0.31006	-1.27869	0.2	[39]
	palladium(II) acetylacetonate	4	0.827-0.865	307.15-333.15	-1.78235	-1.21953	0.5	[35]
	phenanthrene	9	0.406-0.584	473.40-554.00	0.52281	4.90578	2.4	[31]
	phenylbutazone	9	0.828-0.876	298.15-333.15	-3.31207	-1.49768	2	[40]
	2-phenylethyl acetate	5	0.839-0.881	313.16-333.16	-1.03589	-1.03165	3.26	[34]
	3-phenylpropyl acetate	5	0.839-0.881	313.16-333.16	-0.77846	-0.42988	4.63	[34]
	propane	4	0.800-0.884	298.15-348.15	-0.80476	-2.17026	2.69	[24]
	propene	5	0.800-0.884	298.15-348.15	-0.62799	-1.87207	3.13	[24]
	quercetin	9	0.830-0.883	303.15-333.15	-2.34905	-0.79172	5.28	[41]
	toluene	5	0.406-0.774	373.20-554.00	0.01548	0.19645	6.33	[31]
	1,3,5-trimethylbenzene	4	0.406-0.774	373.20-554.00	-0.42827	-0.76163	6.51	[31]
	vanadyl(II) acetylacetonate	4	0.827-0.865	307.15-333.15	-1.74794	-1.40301	1.01	[35]
	water	4	0.767-0.906	278.20-373.20	-3.85108	-2.30682	3.09	[23,32,42]
ethyl acetate	astaxanthin	4	0.838-0.895	303.15-333.15	-0.20068	0.33324	0.85	[43]
	quercetin	5	0.838-0.899	303.15-333.15	-0.22279	0.38481	0.49	[41]
	squalene	5	0.838-0.899	303.15-333.15	-0.18141	-0.00837	0.83	[43]
ethylene glycol	propene	5	1.025-1.084	298.15-348.20	0.13158	8.45657	1.53	[25,26]
methanol	[Bmim][bti]	5	0.841-0.912	283.00-333.00	-0.27284	-0.57579	3.24	[44,45]
	[Emim][bti]	5	0.841-0.912	283.00-333.00	-0.38873	-0.74305	2.79	[44,45]
	[Hmim][bti]	6	0.841-0.912	283.00-333.00	-0.42124	-0.70958	1.42	[45]
	[Omim][bti]	9	0.841-0.912	283.00-333.00	-0.44843	-0.72057	1.15	[45]

Solvent	Solute	NDP	$\rho_{n,1}^{*}$ range	T range (K)	k_{12}	B_{12}	AARD (%)	Data source
	acetonitrile	11	0.868-1.037	283.20-313.20	0.63237	26.43757	6.32	[46]
	acetylferrocene	6	0.839-0.868	312.90-333.40	-0.27697	0.08106	0.38	[29]
	ammonia	6	0.811-0.897	298.15-348.15	-1.06661	-2.15019	5.9	[24]
	benzene	4	0.592-0.870	313.20-473.40	-0.17357	-0.72611	2	[47]
	carbon dioxide	9	0.811-0.897	298.15-348.15	0.10684	-0.31895	2.66	[24]
	carbon monoxide	11	0.797-0.890	298.85-363.15	0.58176	43.18334	3.59	[48]
	p-chloronitrobenzene	13	0.829-0.866	323.20-343.20	-0.31467	-0.89839	0.8	[49]
	disperse blue 14	6	0.849-0.875	308.20-328.20	-0.75549	-0.93907	0.62	[37]
	disperse orange 11	4	0.849-0.875	308.20-328.20	-0.79002	-0.99818	1.11	[37]
	naphthalene	4	0.592-0.870	313.20-473.40	-0.21867	-0.66330	2.92	[47]
	phenanthrene	4	0.592-0.870	313.20-473.40	-0.29778	-0.70599	2.97	[47]
	propane	4	0.811-0.898	298.15-348.15	0.11309	1.02890	2.06	[24]
	toluene	4	0.592-0.870	313.20-473.40	-0.14344	-0.60799	2.7	[47]
	1,3,5-trimethylbenzene	10	0.592-0.870	313.20-473.40	-0.23615	-0.76164	2.7	[47]
	vitamin K_3	10	0.868-0.868	313.20-313.20	0.03769	0.48572	0.45	[50]
	water	5	0.870-0.919	278.20-313.15	-1.19711	-1.50370	1.48	[51]
1-propanol	ammonia	5	0.832-0.910	298.15-348.15	-3.65824	-3.81134	6.09	[24]
	carbon dioxide	10	0.834-0.911	298.15-348.15	-0.71701	-2.54500	2.75	[24]
	propane	5	0.832-0.909	298.15-348.15	-1.06376	-2.80502	3.27	[24]
	propene	5	0.832-0.910	298.15-348.15	-0.94533	-2.69304	2.79	[24]
	water	7	0.847-0.903	298.15-338.15	-5.90091	-2.70509	4.26	[52]
2-propanol	benzene	48	0.430-0.771	373.20-536.00	-0.65622	-1.17026	11.57	[47]

Solvent	Solute	NDP	$\rho^*_{\mathrm{n},1}$ range	T range (K)	k_{12}	B_{12}	AARD (%)	Data source
	n-decane	10	0.430-0.830	333.20-536.00	-1.04297	-1.24802	10.29	[47]
	naphthalene	8	0.430-0.771	373.20-536.00	-0.80790	-0.95772	11.24	[47]
	phenanthrene	7	0.430-0.771	373.20-536.00	-0.86111	-0.83019	8.89	[47]
	n-tetradecane	7	0.430-0.771	373.20-536.00	-0.83255	-0.96523	8.97	[47]
	toluene	7	0.430-0.771	373.20-536.00	-0.65698	-1.09578	11.13	[47]
	water	4	0.819-0.879	298.15-338.15	-7.03715	-2.55482	1.75	[52]

Table S3. Water solvent systems studied, number of data point (NDP), reduced number density range ($\rho^*_{n,1}$ range), absolute temperature range (T range), binary interaction parameters (k_{12} and B_{12}), average absolute relative deviations (AARD) and source of the diffusion data.

Solvent	Solute	NDP	$\rho^*_{n,1}$ range	T range (K)	k_{12}	B_{12}	AARD (%)	Data source
**Water systems**								
water	[Bmim][BF_4]	11	0.973-1.010	283.00-333.00	0.52865	1.36518	0.91	[45,53]
	[Bmim][bti]	18	0.973-1.010	283.00-333.00	0.52815	1.09666	4.09	[45,54,55]
	[Bmim][CF_3SO_3]	5	0.982-0.998	303.20-323.20	0.60840	5.98941	1.31	[53]
	[Bmim][Cl]	6	0.973-1.010	283.00-333.00	0.50863	0.56736	0.85	[45]
	[Bmim][$MeSO_4$]	5	0.982-0.998	303.20-323.20	0.71478	14.25897	1.19	[53]
	[Bmim][$OcSO_4$]	6	0.990-1.008	288.15-313.15	0.50771	0.24793	3.69	[55]
	[Bmim][PF_6]	5	0.982-0.998	303.20-323.20	0.54719	1.62941	1.28	[53]
	[Emim][BF_4]	5	0.982-0.998	303.20-323.20	0.57725	4.07603	1.38	[54]
	[Emim][bti]	18	0.973-1.010	283.00-333.00	0.53195	1.16066	3.52	[44,45,55]
	[Emim][$C_2H_5SO_4$]	11	0.982-1.008	288.15-323.20	0.53970	2.06441	2.83	[54,55]
	[Emim][C_2N_3]	5	0.982-0.998	303.20-323.20	0.52456	1.20970	0.38	[54]
	[Emim][CF_3SO_3]	5	0.982-0.998	303.20-323.20	0.47108	0.06212	1.62	[54]
	[Emim][$MDEGSO_4$]	5	0.982-0.998	303.20-323.20	0.50173	0.80939	2.41	[54]
	[Hmim][bti]	6	0.973-1.010	283.00-333.00	0.54010	1.40123	1.98	[45]
	[Omim][bti]	6	0.973-1.010	283.00-333.00	0.58356	2.78023	2.36	[45]
	acetamide	4	0.992-1.013	277.15-310.15	0.11296	-4.32983	4.67	[56]
	acetone	4	0.977-1.001	298.15-328.15	0.40628	1.77907	0.93	[23]
	alanine	6	0.973-1.004	293.20-333.20	0.16576	-1.91976	0.86	[57]
	alloisoleucine	6	0.973-1.004	293.20-333.20	0.26028	-1.02208	0.58	[57]
	allothreonine	6	0.973-1.004	293.20-333.20	0.22652	-1.22615	0.58	[58]
	ammonia	5	0.973-1.004	293.00-333.00	-0.02810	-6.12836	2.74	[59]
	arabinose	6	0.954-1.015	273.20-353.20	0.27140	-0.65475	2.52	[60]

Solvent	Solute	NDP	$\rho_{n,1}^{*}$ range	*T* range (K)	k_{12}	B_{12}	AARD (%)	Data source
	argon	8	0.994-1.015	273.20-308.00	-0.42718	-17.59434	2.92	[61]
	benzene	24	0.935-1.014	275.15-372.00	0.29281	-1.83559	4.62	[62–65]
	benzoic acid	12	0.969-1.010	283.15-338.15	0.30948	-1.53658	1.11	[66]
	biphenyl	7	0.935-1.013	278.20-372.50	0.35362	-0.55432	1.64	[65]
	n-butane	16	0.973-1.013	277.15-333.15	0.06315	-5.71001	7.3	[62,67,68]
	1,2-butanediol	5	0.935-1.013	278.20-372.50	0.27822	-1.12415	5.16	[32]
	1,3-butanediol	5	0.959-1.001	298.00-348.00	0.25840	-1.22577	1.07	[69]
	1,4-butanediol	5	0.935-1.013	278.20-372.50	0.27474	-1.14167	4.44	[32]
	1-butanol	13	0.935-1.013	277.15-372.00	0.24445	-1.85130	4.52	[56,70,71]
	2-methyl-1-butanol	6	0.954-1.015	273.20-353.20	0.24630	-2.35907	4.28	[72]
	3-methyl-1-butanol	6	0.954-1.015	273.20-353.20	0.24301	-2.29088	4.62	[72]
	2-methyl-2-butanol	6	0.954-1.015	273.20-353.20	0.22374	-2.54516	4.92	[72]
	3-methyl-2-butanol	6	0.954-1.015	273.20-353.20	0.24891	-2.22753	4.59	[72]
	n-butylbenzene	7	0.935-1.013	278.20-372.50	0.30745	-1.14779	1.93	[65]
	α-amino-*n*-butyric acid	6	0.973-1.004	293.20-333.20	0.23475	-1.42859	0.71	[57]
	caffeine	22	0.969-1.004	293.15-338.15	0.33250	-0.28458	0.92	[73–75]
	camphor	8	0.986-1.010	283.15-318.15	0.32226	-0.96978	1.46	[66]
	carbon dioxide	111	0.940-1.015	273.20-368.00	0.17358	-6.06031	5.17	[59,61,67,68,76–80] [81–89][90–98]
	cinnamic acid	8	0.986-1.010	283.15-318.15	0.29408	-2.60915	1.62	[66]
	α-cyclodextrin	4	0.991-1.001	298.15-312.15	0.34724	0.43247	1.32	[99]
	β-cyclodextrin	4	0.991-1.001	298.15-312.15	0.32190	0.14803	1.87	[100]
	cyclohexane	10	0.973-1.014	275.15-333.15	0.24249	-2.57607	6.56	[62,63]
	cyclopentane	10	0.973-1.014	275.15-333.15	0.21202	-2.81080	2.95	[62,63]
	diethanolamine	5	0.959-1.001	298.00-348.00	0.29822	-0.60829	0.68	[101]
	diethylene glycol	5	0.982-0.998	303.20-323.20	0.31092	-0.36908	0.87	[102]

Solvent	Solute	NDP	$\rho_{n,1}^{*}$ range	T range (K)	k_{12}	B_{12}	AARD (%)	Data source
	N,*N*-diethylethanolamine	5	0.962-0.996	303.15-343.15	0.28693	-0.42148	0.41	[103,104]
	diglycolamine	5	0.964-0.998	303.15-343.15	0.30147	-0.25730	0.28	[105]
	diisopropanolamine	5	0.959-1.001	298.00-348.00	0.27630	-0.74476	2.58	[101]
	dipropylene glycol	5	0.982-0.998	303.20-323.20	0.39347	2.28996	0.63	[106]
	dimethylethanolamine (DMEA)	5	0.962-0.996	303.15-343.15	0.28085	0.00423	1.45	[103,104,107]
	meso-erythritol	5	0.935-1.013	278.20-372.50	0.25912	-0.67481	3.99	[32]
	ethane	16	0.973-1.013	277.15-333.15	0.12384	-6.28928	6.87	[62,67,108]
	ethanol	22	0.935-1.009	285.65-372.00	0.26134	-1.74453	3.44	[23,42,56,70,71]
	ethylbenzene	16	0.939-1.014	275.15-368.20	0.31913	-0.82257	4.32	[62–64]
	ethylene glycol	9	0.954-1.001	298.20-353.20	0.24859	-1.47686	1.52	[71,102]
	1-ethylnaphthalene	7	0.935-1.013	278.20-372.50	0.32914	-0.69771	2.02	[65]
	formamide	4	0.992-1.013	277.15-310.15	0.20577	-3.80312	5.41	[56]
	fructose	4	0.991-1.001	298.15-312.15	0.27614	0.30135	0.52	[109]
	furfural	7	0.954-1.001	298.20-353.20	0.35442	-0.70268	1.53	[110]
	5-(hydroxymethyl) furfural	7	0.954-1.001	298.20-353.20	0.33463	-0.53108	1.01	[110]
	galactose	6	0.954-1.015	273.20-353.20	0.28446	-0.48122	2.48	[60]
	glucose	10	0.954-1.015	273.20-353.20	0.23420	-0.87311	1.67	[60,109]
	glycerol	10	0.954-1.013	278.20-353.20	0.25979	-1.19987	4.03	[69,71]
	glycine	6	0.973-1.004	293.20-333.20	0.26553	-1.28508	1.05	[57]
	guaiacol	7	0.954-1.001	298.20-353.20	0.32076	-0.81619	0.51	[110]
	hexafluorobenzene	6	0.939-1.001	298.20-368.20	0.30543	-0.77687	2.46	[64]
	1,2,6-hexanetriol	5	0.959-1.001	298.00-348.00	0.29757	-0.43831	1.72	[69]
	homoserine	6	0.973-1.004	293.20-333.20	0.25430	-1.09473	0.6	[58]
	hydrogen	6	0.994-1.010	283.15-308.15	-2.15700	-97.73803	9.51	[68,111]
	hydrogen sulfide	22	0.940-1.008	288.15-368.00	0.31355	-1.36227	3.8	[67,95,112]

Solvent	Solute	NDP	$\rho_{n,1}^{*}$ range	T range (K)	k_{12}	B_{12}	AARD (%)	Data source
	myo-inositol	5	0.935-1.013	278.20-372.50	0.24881	-0.67604	1.99	[32]
	isobutyramide	4	0.992-1.013	277.15-310.15	0.12798	-4.47613	6.12	[56]
	isoleucine	6	0.973-1.004	293.20-333.20	0.25412	-1.14983	0.6	[57]
	krypton	6	0.994-1.010	283.15-308.15	-0.03806	-11.64743	1.22	[68,111]
	lactose	5	0.986-1.001	298.15-318.15	0.31736	0.34812	0.74	[109]
	leucine	6	0.973-1.004	293.20-333.20	0.25166	-1.16495	0.79	[57]
	tert-leucine	6	0.973-1.004	293.20-333.20	0.23863	-1.31213	0.9	[57]
	mannitol	5	0.935-1.013	278.20-372.50	0.26271	-0.44580	2	[32]
	mannose	6	0.954-1.015	273.20-353.20	0.27583	-0.65285	2.01	[60]
	monoethanolamine (MEA)	9	0.962-1.001	298.15-343.15	0.36833	0.31345	3.38	[101,104,107]
	methane	32	0.964-1.013	278.00-342.80	0.01652	-11.08869	5.36	[61,62,67,108]
	methanol	15	0.944-1.009	285.65-363.15	0.20075	-3.25337	6.14	[51,56,113–115]
	methyl bromide	6	0.994-1.010	283.00-308.00	-0.03792	-7.28746	1.11	[61]
	methyl chloride	6	0.994-1.010	283.00-308.00	-0.18564	-9.19829	1.15	[61]
	methyl fluoride	6	0.994-1.010	283.00-308.00	-0.33512	-12.92964	3.72	[61]
	2-methyl-2,4-pentanediol	5	0.959-1.001	298.00-348.00	0.26789	-1.03766	0.61	[69]
	methylcyclopentane	10	0.973-1.014	275.15-333.15	0.23407	-2.14456	3.74	[62,63]
	n-methyldiethanolamine	5	0.959-1.001	298.00-348.00	0.27047	-0.92842	0.73	[101]
	N-methylpyrrolidone	5	0.959-1.001	298.00-348.00	0.32520	-0.65032	0.76	[69]
	monoisopropanolamine (MIPA)	5	0.962-0.996	303.15-343.15	0.22028	-1.51899	1.1	[104,107]
	naphthalene	7	0.935-1.013	278.20-372.50	0.34537	-0.88326	2.32	[65]
	2-naphthol	16	0.939-1.010	283.15-368.15	0.39722	-0.69114	2.79	[66]
	neon	6	0.994-1.010	283.15-308.15	0.00799	-35.02102	1.11	[68,111]
	nitrogen	10	0.893-1.020	298.00-423.00	0.46340	6.57895	7.43	[97]
	nitrous oxide	78	0.939-1.008	288.00-368.15	0.16846	-6.01209	7.55	[95,96,116]

Solvent	Solute	NDP	$\rho_{n,1}^{*}$ range	T range (K)	k_{12}	B_{12}	AARD (%)	Data source
	norleucine	6	0.973-1.004	293.20-333.20	0.26051	-1.02458	0.58	[57]
	norvaline	6	0.973-1.004	293.20-333.20	0.24313	-1.28928	0.43	[57]
	oxygen	34	0.939-1.011	282.35-368.15	0.18458	-6.53108	5.63	[28,117]
	pentaerythritol	6	0.935-1.013	278.20-372.20	0.28780	-0.50717	3.03	[71]
	n-pentane	8	0.973-1.013	277.15-333.15	0.23099	-3.22012	7.12	[62,67]
	1-pentanol	6	0.954-1.015	273.20-353.20	0.23776	-2.51764	4.85	[72]
	2-pentanol	6	0.954-1.015	273.20-353.20	0.26187	-2.06253	5.13	[72]
	3-pentanol	6	0.954-1.015	273.20-353.20	0.24794	-2.30725	4.56	[72]
	phenol	17	0.502-1.012	298.20-648.42	0.42691	2.02936	10.77	[110,118]
	2-piperidineethanol	5	0.964-0.998	303.15-343.15	0.29065	-0.43556	0.51	[105]
	potassium chloride	30	0.880-1.028	298.00-423.15	0.65304	0.40267	15.26	[101,103,119]
	propanamide	4	0.992-1.013	277.15-310.15	0.12900	-4.39181	6.38	[56]
	propane	16	0.973-1.013	277.15-333.15	0.11701	-5.77803	6.67	[62,67,108]
	1-propanol	15	0.935-1.013	277.15-372.00	0.23117	-2.15789	5.86	[52,56,71]
	2-amino-2-methyl-1-propanol	5	0.964-0.998	303.15-343.15	0.29178	-0.59819	0.75	[105]
	2,2-dimethyl-1-propanol	6	0.954-1.015	273.20-353.20	0.24439	-2.37654	5.15	[72]
	2-propanol	12	0.953-1.013	277.15-354.15	0.23226	-2.24662	6.62	[52,56,114]
	2-methyl-2-propanol	9	0.935-1.013	278.20-372.50	0.20785	-2.22607	7.01	[71]
	propylene glycol	5	0.982-0.998	303.20-323.20	0.26334	-1.37822	0.68	[106]
	piperazine (PZ)	5	0.962-0.996	303.15-343.15	0.30426	0.24249	1.3	[104,107]
	radon	6	0.994-1.010	283.15-308.15	-0.03671	-7.01665	1.27	[68]
	salicylic acid	13	0.964-1.010	283.15-343.15	0.35493	-1.27324	1.31	[66]
	serine	6	0.973-1.004	293.20-333.20	0.22863	-1.30671	0.56	[58]
	sucrose	10	0.954-1.015	273.20-353.20	0.31231	0.10757	1.79	[60,109]
	sulfolane	5	0.962-0.996	303.15-343.15	0.35029	-0.31336	0.96	[104]

Solvent	Solute	NDP	$\rho_{n,1}^{*}$ range	T range (K)	k_{12}	B_{12}	AARD (%)	Data source
	sulfur dioxide	4	0.994-1.004	293.15-308.15	0.21996	-4.87093	0.32	[86]
	tetraethylene glycol	5	0.982-0.998	303.20-323.20	0.31313	0.40270	0.61	[102]
	threonine	6	0.973-1.004	293.20-333.20	0.22262	-1.15207	0.72	[58]
	toluene	15	0.939-1.014	275.15-368.20	0.27118	-1.84101	5.72	[62–64]
	triethanolamine	5	0.964-0.998	303.15-343.15	0.30469	-0.19831	0.32	[105]
	triethylene glycol	5	0.982-0.998	303.20-323.20	0.36084	1.57284	0.35	[102]
	valine	6	0.973-1.004	293.20-333.20	0.23744	-1.35663	0.68	[57]
	vanillin	7	0.954-1.001	298.20-353.20	0.31369	-0.68948	0.49	[110]
	xenon	6	0.994-1.010	283.15-308.15	-0.05515	-8.98504	1.31	[68,111]
	xylitol	5	0.935-1.013	278.20-372.50	0.27673	-0.53829	2.45	[32]
	xylose	6	0.954-1.015	273.20-353.20	0.26993	-0.69936	3.13	[60]

Table S4. Non-polar solvent systems studied, number of data point (NDP), reduced number density range ($\rho^*_{n,1}$ range), absolute temperature range (T range), binary interaction parameters (k_{12} and B_{12}), average absolute relative deviations (AARD) and source of the diffusion data.

Solvent	Solute	NDP	$\rho^*_{n,1}$ range	T range (K)	k_{12}	B_{12}	AARD (%)	Data source
**Non-Polar systems**								
cyclohexane	acetone	4	0.846-0.886	303.20-333.20	-0.99998	-1.76875	0.26	[120]
	argon	7	0.707-0.879	298.15-415.85	-0.94976	-4.14846	2.28	[121]
	benzene	12	0.503-0.886	298.20-523.20	-0.97887	-1.36443	2.62	[120,122]
	carbon tetrachloride	7	0.707-0.879	298.15-415.85	-1.08414	-1.26220	1.61	[121]
	1,1′-dimethylferrocene	5	0.857-0.876	313.15-323.15	-1.11714	-0.95520	3.25	[123]
	ethane	5	0.784-0.909	280.50-363.20	-0.83291	-2.31941	1.28	[124]
	ethylene	5	0.784-0.909	280.50-363.20	-0.81406	-2.36780	0.73	[124]
	ethylferrocene	6	0.857-0.876	313.15-323.15	-1.04914	-0.79760	0.74	[123]
	ferrocene	5	0.857-0.876	313.15-323.15	-1.11665	-0.74016	3.27	[123]
	krypton	6	0.707-0.858	313.25-415.85	-0.72136	-2.23326	0.88	[121]
	methane	6	0.707-0.858	313.25-415.85	-0.91351	-3.91996	0.57	[121]
	naphthalene	12	0.503-0.886	298.20-523.20	-0.91881	-0.98873	1.84	[120,122]
	phenanthrene	8	0.503-0.879	298.20-523.20	-1.00226	-0.87699	2.7	[122]
	tetrabutyltin	7	0.707-0.879	298.15-415.85	-1.59017	-1.07932	2.17	[121]
	tetraethyltin	7	0.707-0.879	298.15-415.85	-1.29726	-1.13906	1.52	[121]
	tetramethyltin	7	0.707-0.879	298.15-415.85	-1.20779	-1.31927	1.61	[121]
	tetrapropyltin	6	0.707-0.879	298.15-415.85	-1.35613	-0.99509	1.61	[121]
	toluene	12	0.503-0.886	298.20-523.20	-0.85461	-1.16758	1.69	[120,122]
	1,3,5-trimethylbenzene	12	0.503-0.886	298.20-523.20	-0.97415	-1.13155	2.01	[120,125]
	xenon	7	0.707-0.879	298.15-415.85	-0.85317	-1.78244	1.3	[121]
	m-xylene	4	0.846-0.886	303.20-333.20	-0.90350	-1.14622	1.13	[120]
	p-xylene	8	0.503-0.879	298.20-523.20	-0.79997	-1.07468	2.12	[122]

Solvent	Solute	NDP	$\rho^*_{n,1}$ range	T range (K)	k_{12}	B_{12}	AARD (%)	Data source
n-decane	argon	3	0.783-0.970	298.00-433.00	-0.09651	0.85628	1.88	[126]
	carbon tetrachloride	3	0.865-0.970	298.00-373.00	-0.00520	1.26645	0.17	[126]
	12-crown-4	4	0.864-0.969	298.20-373.20	-0.09776	0.67611	2.34	[127]
	15-crown-5	4	0.864-0.969	298.20-373.20	-0.07705	1.04026	4.5	[127]
	18-crown-6	4	0.864-0.969	298.20-373.20	-0.13023	0.71096	2.06	[127]
	dicyclohexano-18-crown-6	4	0.864-0.969	298.20-373.20	-0.18586	0.46112	0.85	[127]
	dicyclohexano-24-crown-8	4	0.864-0.969	298.20-373.20	-0.21455	0.42683	1.4	[127]
	krypton	3	0.783-0.970	298.00-433.00	-0.09584	0.73720	1.28	[126]
	tetrabutyltin	4	0.783-0.970	298.00-433.00	-0.09901	1.16820	1.97	[126]
	tetraethyltin	4	0.783-0.970	298.00-433.00	0.13031	1.89101	1.75	[126]
	tetramethyltin	4	0.783-0.970	298.00-433.00	-0.03276	1.62518	1.81	[126]
	tetrapropyltin	4	0.783-0.970	298.00-433.00	-0.05994	1.26261	1.58	[126]
	s-trioxane	4	0.864-0.969	298.20-373.20	-0.05371	1.13517	0.45	[127]
	xenon	8	0.783-0.992	283.15-433.00	0.19250	5.47973	3.72	[126,128]
2,3-dimethylbutane	benzene	11	0.401-0.539	523.20-548.20	-0.03258	0.42952	1.24	[129]
	naphthalene	9	0.401-0.539	523.20-548.20	0.03232	0.61984	1.09	[129]
	phenanthrene	11	0.401-0.539	523.20-548.20	0.10585	0.95224	0.96	[129]
	toluene	10	0.401-0.539	523.20-548.20	0.01486	0.62566	1.25	[129]
n-dodecane	acetone	5	0.948-0.999	303.20-343.20	-0.10306	1.24817	1.04	[120]
	benzene	4	0.960-0.999	303.20-333.20	-0.01865	2.38119	0.6	[120]
	carbon dioxide	9	0.629-0.984	304.15-567.15	-0.18406	-0.14044	3.04	[130]
	carbon monoxide	9	0.629-0.984	304.15-567.15	0.04926	6.46420	5.85	[130]
	n-decane	5	0.636-0.984	304.00-566.00	0.03589	1.39604	1.99	[131]
	n-hexadecane	5	0.636-0.984	304.00-566.00	-0.06219	0.94291	3.28	[131]
	linoleic acid methyl ester	4	0.960-0.999	303.20-333.20	-0.12620	1.44332	0.25	[120]

Solvent	Solute	NDP	$\rho_{n,1}^{*}$ range	T range (K)	k_{12}	B_{12}	AARD (%)	Data source
	naphthalene	5	0.948-0.999	303.20-343.20	0.07516	2.67099	1.35	[120]
	n-octane	9	0.636-0.984	304.00-566.00	0.00062	1.04852	1.24	[131]
	n-tetradecane	5	0.636-0.984	304.00-566.00	0.04835	1.69011	5.59	[131]
	toluene	4	0.960-0.999	303.30-333.20	0.06888	3.47694	1.23	[120]
	1,3,5-trimethylbenzene	4	0.960-0.999	303.20-333.20	0.00440	2.41818	0.93	[120]
	vitamin K_3	4	0.960-0.998	303.20-333.20	0.07031	2.00891	0.4	[120]
	m-xylene	4	0.960-0.999	303.20-333.20	0.10711	4.18011	0.88	[120]
n-eicosane	carbon dioxide	5	0.793-0.977	374.25-533.45	-0.08617	2.75972	3.45	[132]
	carbon monoxide	5	0.793-0.977	374.25-533.45	-0.22173	3.16627	4.31	[132]
	n-dodecane	5	0.793-0.977	374.95-533.55	-0.05673	1.38497	3.06	[132]
	n-hexadecane	5	0.793-0.977	374.95-533.55	-0.00752	1.97025	1.97	[132]
	n-octane	5	0.793-0.977	374.95-533.55	-0.06544	1.35955	3.76	[132]
ethane	1-octene	6	0.429-0.562	296.20-322.20	0.76318	26.53913	3.83	[133]
	1-tetradecene	9	0.428-0.570	293.20-322.20	0.33823	1.50634	1.85	[133]
n-heptane	benzene	11	0.853-0.899	303.20-333.20	0.13287	1.68363	2.33	[134,135]
	n-decane	6	0.632-0.906	298.15-477.00	0.18070	1.89752	0.83	[131,136]
	n-dodecane	6	0.632-0.910	298.15-477.00	0.12723	1.41863	1.54	[131,136]
	ethylbenzene	4	0.853-0.899	303.20-333.20	0.20580	2.99935	0.14	[135]
	n-hexadecane	9	0.615-0.906	298.15-477.00	0.00353	0.66485	1.6	[131,137]
	n-hexane	11	0.853-0.930	283.00-333.00	0.14578	1.67072	1.21	[138–140]
	n-octane	13	0.632-0.914	293.00-477.00	0.09397	1.06147	1.57	[131,138,141]
	n-tetradecane	6	0.632-0.910	298.15-477.00	0.14192	1.63129	1.72	[131,136]
	toluene	4	0.853-0.899	303.20-333.20	0.44464	8.44341	0.37	[135]
	1,3,5-trimethylbenzene	4	0.853-0.899	303.20-333.20	0.14830	2.06322	0.53	[135]
	2,2,4-trimethylpentane	4	0.868-0.891	308.15-323.15	0.07215	1.34296	0.26	[141]

Solvent	Solute	NDP	$\rho^*_{n,1}$ range	T range (K)	k_{12}	B_{12}	AARD (%)	Data source
	o-xylene	4	0.853-0.899	303.20-333.20	0.64538	25.10848	0.15	[135]
	p-xylene	4	0.853-0.899	303.20-333.20	0.38684	5.82892	0.46	[135]
n-hexadecane	carbon dioxide	10	0.679-0.986	323.15-564.15	-0.27857	0.20087	2.29	[130]
	carbon monoxide	10	0.679-0.986	323.15-564.15	-0.32920	0.92762	4.57	[130]
	n-decane	5	0.696-0.983	323.15-564.15	-0.27285	-0.31515	1.97	[142]
	n-dodecane	5	0.696-0.983	323.15-564.15	-0.15491	0.47574	1.17	[142]
	n-octane	10	0.696-0.984	323.15-564.15	-0.23865	-0.15544	1.48	[142]
	n-tetradecane	5	0.696-0.983	323.15-564.15	-0.15140	0.50899	2.16	[142]
n-hexane	acetone	5	0.834-0.877	303.20-333.20	0.09082	1.42164	2.07	[120]
	acetonitrile	7	0.863-1.064	298.20-298.20	0.28856	4.96746	6.27	[143]
	benzene	48	0.380-1.058	213.20-543.20	0.17193	1.83003	6.06	[120,124,125,143–147]
	carbon disulfide	10	0.863-1.063	298.20-298.20	0.35216	5.92373	9.86	[143]
	carbon tetrabromide	8	0.864-1.058	298.15-298.15	0.39401	8.09433	22.48	[148]
	o-difluorobenzene	7	0.806-1.005	213.20-333.20	0.20682	2.12792	2.59	[147]
	p-difluorobenzene	7	0.806-1.005	213.20-333.20	0.23316	2.32080	0.7	[147]
	9,10-dimethylanthracene	7	0.864-1.042	298.15-298.15	0.51792	11.50959	14.57	[148]
	1,1′-dimethylferrocene	4	0.839-0.866	313.15-313.15	-0.12998	0.55089	0.2	[123]
	ethylferrocene	4	0.839-0.866	313.15-313.15	-0.15202	0.42538	0.15	[123]
	ferrocene	4	0.839-0.866	313.15-313.15	-0.13382	0.37529	0.2	[123]
	n-heptane	11	0.817-0.888	283.00-398.00	0.00601	0.48172	5.57	[138–140,149]
	hexafluorobenzene	7	0.806-1.005	213.20-333.20	0.30757	4.09154	2.07	[147]
	indole	2	0.861-0.873	313.20-313.20	-0.04069	0.07991	0	[120]
	linoleic acid methyl ester	2	0.861-0.873	313.20-313.20	0.25029	3.51321	0	[120]
	naphthalene	21	0.380-0.875	298.15-543.20	0.02615	0.52119	4.31	[120,125,143,144]
	octafluorotoluene	7	0.806-1.005	213.20-333.20	0.30312	4.06106	2.45	[147]

Solvent	Solute	NDP	$\rho^*_{n,1}$ range	T range (K)	k_{12}	B_{12}	AARD (%)	Data source
	n-octane	7	0.817-0.869	295.00-328.00	0.07885	1.04919	0.36	[138,149]
	pentafluorobenzene	7	0.806-1.005	213.20-333.20	0.33643	4.77299	2.2	[147]
	phenanthrene	15	0.380-0.807	333.20-543.20	0.07098	0.66932	4.57	[125]
	pyrene	8	0.864-1.058	298.15-298.15	0.34687	10.45121	21.89	[144,148]
	1,2,3,5-tetrafluorobenzene	7	0.806-1.005	213.20-333.20	0.29722	3.09569	2.78	[147]
	1,2,4,5-tetrafluorobenzene	7	0.806-1.005	213.20-333.20	0.28385	2.99616	1.91	[147]
	toluene	32	0.380-1.061	298.15-543.20	0.23061	2.50601	7.61	[120,125,150,151]
	1,2,4-trifluorobenzene	7	0.806-1.005	213.20-333.20	0.29651	3.25699	2.67	[147]
	1,3,5-trimethylbenzene	20	0.380-0.875	303.20-543.20	-0.03623	0.40118	3.51	[120,125]
	vitamin K_3	5	0.862-0.873	313.20-313.20	0.77847	50.92952	0.4	[120,152]
	m-xylene	5	0.834-0.877	303.20-333.20	-0.14250	-0.23973	2.18	[120]
	p-xylene	17	0.380-0.873	313.20-543.20	-0.07620	0.14052	5.14	[120,125]
n-octane	argon	4	0.775-0.937	298.00-403.00	0.10348	4.26793	0.91	[126]
	benzene	8	0.885-0.929	303.20-333.20	0.30540	5.01801	0.18	[135,153]
	carbon tetrachloride	4	0.827-0.937	298.00-373.00	0.11638	1.83525	0.43	[126]
	ethylbenzene	8	0.885-0.929	303.20-333.20	0.47833	13.09162	1.47	[135,153]
	n-heptane	7	0.871-0.945	293.00-343.00	0.21198	2.77075	0.73	[138,154]
	n-hexane	6	0.885-0.942	295.00-333.00	-0.00023	0.16762	0.86	[138]
	krypton	4	0.775-0.937	298.00-403.00	0.07118	2.54908	0.71	[126]
	methane	4	0.775-0.937	298.00-403.00	-0.10515	0.66788	1.18	[126]
	tetrabutyltin	4	0.739-0.937	298.00-433.00	-0.04388	0.93694	1.4	[126]
	tetraethyltin	5	0.739-0.937	298.00-433.00	0.04782	1.36630	4.84	[126]
	tetramethyltin	4	0.739-0.937	298.00-433.00	0.05732	1.79520	0.36	[126]
	tetrapropyltin	4	0.739-0.937	298.00-433.00	-0.01033	1.04338	0.46	[126]
	toluene	8	0.885-0.929	303.20-333.20	0.52387	15.43289	1.42	[135,153]

Solvent	Solute	NDP	$\rho_{n,1}^{*}$ range	T range (K)	k_{12}	B_{12}	AARD (%)	Data source
	1,3,5-trimethylbenzene	8	0.885-0.929	303.20-333.20	0.12676	2.13992	0.43	[135,153]
	xenon	8	0.775-0.959	283.15-403.00	0.39781	10.76050	4.89	[126,128]
	o-xylene	8	0.885-0.929	303.20-333.20	0.16370	2.07088	0.82	[135,153]
	p-xylene	8	0.885-0.929	303.20-333.20	0.16241	1.62376	0.69	[135,153]
propane	1-octene	8	0.585-0.697	296.50-337.50	0.31072	2.51472	1.59	[133]
	1-tetradecene	8	0.606-0.705	292.50-337.40	0.81421	43.88873	1.76	[133]
sulfur hexafluoride	benzene	9	0.116-0.539	328.00-328.00	0.03318	0.74521	6.24	[27]
	benzoic acid	6	0.352-0.539	328.20-338.20	-0.11493	0.42950	1.84	[155]
	carbon tetrachloride	7	0.116-0.539	328.00-328.00	-0.19119	0.12578	7.52	[27]
	naphthalene	5	0.427-0.576	318.20-328.20	-0.46893	-0.53952	5.79	[155]
	toluene	11	0.116-0.539	328.00-328.00	0.17557	1.49396	5.46	[27]
	1,3,5-trimethylbenzene	10	0.193-0.539	328.00-328.00	0.16632	1.40391	3.74	[27]
	p-xylene	52	0.116-0.650	283.20-338.15	-0.00033	0.50720	5.68	[27]
n-tetradecane	acridine	8	0.801-0.970	333.20-473.15	0.01804	2.24474	4.08	[156]
	argon	4	0.854-1.013	298.00-430.00	0.21924	16.93113	3.11	[126]
	benzothiophene	7	0.826-0.970	333.20-453.20	-0.33312	0.09757	2.36	[156]
	carbon tetrachloride	4	0.921-1.013	298.00-374.00	-0.04611	2.90522	0.58	[126]
	dibenzothiophene	8	0.813-0.970	333.20-463.20	-0.22664	0.56996	4.1	[156]
	krypton	4	0.854-1.013	298.00-430.00	0.13416	7.91996	2.41	[126]
	methane	4	0.854-1.013	298.00-430.00	-0.23313	2.26854	7.85	[126]
	naphthalene	7	0.826-0.995	313.20-453.20	-0.17348	0.35564	3.32	[156]
	tetrabutyltin	4	0.854-1.013	298.00-430.00	-0.22454	1.28179	1.62	[126]
	tetraethyltin	4	0.854-1.013	298.00-430.00	-0.13720	1.63681	1.29	[126]
	tetramethyltin	4	0.854-1.013	298.00-430.00	-0.18410	1.36224	0.95	[126]
	tetrapropyltin	4	0.854-1.013	298.00-430.00	-0.16872	1.40527	0.71	[126]

Solvent	Solute	NDP	$\rho_{n,1}^{*}$ range	T range (K)	k_{12}	B_{12}	AARD (%)	Data source
	xenon	8	0.854-1.032	283.15-430.00	0.06084	4.54353	5.15	[126,128]
2,2,4-trimethylpentane	benzene	4	0.651-0.684	303.20-333.20	0.54031	25.73093	5	[153]
	1,3,5-trimethylbenzene	4	0.651-0.684	303.20-333.20	0.68248	56.85121	7.7	[153]
	ethylbenzene	4	0.651-0.684	303.20-333.20	0.63060	39.48716	3.65	[153]
	toluene	4	0.651-0.684	303.20-333.20	0.57565	28.01625	4.39	[153]
	o-xylene	4	0.651-0.684	303.20-333.20	0.82761	178.58484	5.86	[153]
	p-xylene	4	0.651-0.684	303.20-333.20	0.74454	72.40594	2.68	[153]

Table S5. SC-CO_2 solvent systems studied, number of data point (NDP), reduced number density range ($\rho^*_{n,1}$ range), absolute temperature range (T range), binary interaction parameters (k_{12} and B_{12}), average absolute relative deviations (AARD) and source of the diffusion data.

Solvent	Solute	NDP	$\rho^*_{n,1}$ range	T range (K)	k_{12}	B_{12}	AARD (%)	Data source
**SC-CO_2 systems**								
carbon dioxide	acetone	213	0.218-0.589	303.15-333.15	0.04553	0.32822	3.84	[157–161]
	acetylferrocene	24	0.251-0.472	313.00-372.70	-0.23523	-0.16124	4.67	[29]
	acridine	6	0.426-0.555	308.15-328.15	0.45449	2.83796	1.26	[162]
	adamantanone	8	0.376-0.470	313.45-313.45	0.38365	1.33278	2.29	[163]
	allylbenzene	15	0.363-0.565	313.16-333.16	0.40934	2.97617	1.9	[164]
	aluminum acetylacetonate	84	0.201-0.588	308.15-333.15	0.61164	10.95789	7.7	[30]
	aniline	15	0.363-0.541	313.16-333.16	0.38735	3.65582	2.49	[165]
	anisole	15	0.363-0.565	313.00-333.00	0.12107	0.73485	1.93	[166]
	anthracene	22	0.215-0.564	313.00-333.00	0.02025	0.10628	0.44	[167]
	arachidonic acid (AA)	75	0.331-0.564	308.15-343.15	0.41685	2.00746	2.47	[168]
	AA ethyl ester	48	0.299-0.514	308.15-338.15	0.12009	0.34741	0.92	[169]
	behenic acid ethyl ester	17	0.361-0.514	308.00-318.00	0.07806	0.16505	0.67	[170]
	benzene	249	0.168-0.565	303.15-333.15	0.38669	2.41159	6.8	[158,171–178]
	benzoic acid	35	0.324-0.555	293.15-328.15	0.22502	1.03677	5.24	[162,179–181]
	benzyl acetate	15	0.363-0.565	313.16-333.16	0.13810	0.77642	1.91	[34]
	benzylacetone	15	0.363-0.565	313.16-333.16	0.20190	1.07523	2.06	[182]
	biphenyl	24	0.324-0.556	293.15-323.15	0.46807	2.76765	2.91	[181]
	2-bromoanisole	15	0.363-0.565	313.16-333.16	0.13949	0.83615	1.93	[164]
	bromobenzene	21	0.363-0.565	313.00-333.00	0.05687	0.36678	4.35	[183,184]
	2-butanone	40	0.352-0.575	308.15-328.15	0.10700	0.52037	1.6	[159,184,185]
	N-(4-methoxybenzylidene)-4-*n*-butylaniline	5	0.432-0.479	313.45-313.45	-0.09218	-0.15264	0.23	[163]

Solvent	Solute	NDP	$\rho^*_{n,1}$ range	T range (K)	k_{12}	B_{12}	AARD (%)	Data source
	n-butylbenzene	15	0.363-0.565	313.00-333.00	0.22796	1.06094	1.62	[186]
	sec-butylbenzene	15	0.363-0.565	313.00-333.00	0.11977	0.59033	1.9	[13]
	tert-butylbenzene	15	0.363-0.565	313.00-333.00	-0.01652	0.07490	3.02	[187]
	butyric acid ethyl ester	16	0.361-0.514	308.00-318.00	-0.12516	-0.14767	1.89	[188,189]
	caffeine	25	0.260-0.489	308.00-333.15	0.71487	13.32417	4.15	[190–192]
	capric acid ethyl ester	16	0.361-0.514	308.00-318.00	-0.02908	0.01267	1.26	[188,189]
	caprylic acid ethyl ester	16	0.361-0.514	308.00-318.00	-0.04902	-0.00440	1.5	[188,189]
	β-carotene	90	0.376-0.564	308.15-333.15	0.41649	1.51020	0.94	[193–195]
	L-carvone	27	0.388-0.563	308.15-338.15	-0.24023	-0.27104	2.98	[196,197]
	chlorobenzene	21	0.363-0.565	313.00-333.00	0.08985	0.52518	3.19	[183,184]
	chromium(III) acetylacetonate	104	0.240-0.589	308.15-343.15	0.30738	1.71763	4.15	[36,198]
	chrysene	4	0.478-0.534	303.15-333.15	0.29031	0.92354	0.67	[158]
	citral	15	0.267-0.509	313.15-333.15	0.14094	0.55670	2.51	[199]
	cobalt(III) acetylacetonate	38	0.362-0.578	313.15-333.15	0.04975	0.66805	1.39	[200]
	copper(II) trifluoroacetylacetonate	12	0.363-0.499	308.15-318.15	0.14910	1.78495	4.43	[201]
	15-crown-5	29	0.255-0.550	308.18-313.20	0.26549	0.97552	4.53	[202]
	dibenzo-24-crown-8	28	0.471-0.574	308.18-313.20	0.09150	0.22837	1.6	[202]
	cycloheptanone	8	0.367-0.491	314.15-314.15	-0.25564	-0.16238	1.58	[203]
	cyclononanone	8	0.367-0.491	314.15-314.15	-0.18683	-0.06842	2.21	[203]
	cyclopentanone	8	0.367-0.491	314.15-314.15	-0.00714	0.31969	0.9	[203]
	n-decane	5	0.442-0.497	299.15-308.15	-0.31529	-0.67361	1.63	[204]
	dibenzyl ether	15	0.363-0.565	313.16-333.16	0.29618	1.65277	1.63	[34]
	1,2-dichlorobenzene	15	0.363-0.565	313.00-333.00	0.29190	1.58648	0.81	[187]
	1,3-dichlorobenzene	4	0.472-0.527	313.15-313.15	-0.35663	-0.36790	1.07	[196]

Solvent	Solute	NDP	$\rho^*_{n,1}$ range	T range (K)	k_{12}	B_{12}	AARD (%)	Data source
	p-dichlorobenzene	13	0.324-0.532	298.15-318.15	0.20814	1.09424	3.27	[181]
	diethyl ether	17	0.117-0.482	313.15-333.15	0.69641	18.14313	9.65	[184,185,205]
	1,2-diethylbenzene	15	0.363-0.565	313.16-333.16	0.22735	1.07764	1.69	[206]
	1,4-diethylbenzene	15	0.363-0.565	313.16-333.16	0.05375	0.30396	2.39	[206]
	diisopropyl ether	15	0.117-0.482	313.15-333.15	0.72561	19.64632	7.39	[205]
	2,3-dimethylaniline	15	0.363-0.565	313.00-333.00	0.06943	0.63108	2.17	[207]
	2,6-dimethylaniline	15	0.363-0.565	313.00-333.00	0.27891	1.69492	1.93	[207]
	1,1′-dimethylferrocene	68	0.233-0.589	308.15-323.15	0.05949	0.57610	2.32	[208]
	2,3-dimethylnaphthalene	3	0.429-0.521	308.20-308.20	0.13116	0.50696	0.92	[209]
	2,6-dimethylnaphthalene	6	0.405-0.522	308.20-308.20	0.22069	0.87406	3.71	[209,210]
	2,7-dimethylnaphthalene	6	0.445-0.524	308.20-308.20	0.14828	0.60017	4.32	[209,210]
	2,4-dimethylphenol	15	0.363-0.565	313.00-333.00	0.30169	1.87922	1.95	[166]
	diolein	9	0.380-0.531	313.21-313.21	0.40597	1.56624	1.46	[211]
	disperse blue 14	47	0.496-0.561	310.00-320.00	0.32475	0.96539	2.42	[37]
	disperse orange 11	65	0.474-0.577	308.20-328.20	0.05402	0.05804	3.41	[37]
	1,3-divinylbenzene	15	0.363-0.565	313.16-333.16	0.18206	0.84320	1.19	[164]
	docosahexaenoic acid (DHA)	63	0.340-0.563	308.15-343.15	0.19423	0.79944	2.03	[212]
	DHA ethyl ester	65	0.299-0.514	308.00-338.15	0.13539	0.41781	1.58	[169,170]
	DHA methyl ester	17	0.361-0.514	308.00-318.00	0.08781	0.26123	0.71	[170]
	n-dodecane	5	0.442-0.497	299.15-308.15	-0.50174	-0.76576	3.16	[204]
	eicosapentaenoic acid (EPA)	55	0.323-0.553	308.15-343.15	0.27696	1.01661	2.33	[212]
	EPA ethyl ester	48	0.299-0.514	308.15-338.15	0.08947	0.26949	0.92	[169]
	EPA methyl ester	17	0.361-0.514	308.00-318.00	0.03397	0.12162	0.44	[170]
	ethanol	24	0.350-0.531	313.21-313.21	0.24065	1.46890	2.71	[179]
	ethyl acetate	16	0.129-0.489	308.00-328.00	0.71495	19.67313	13.58	[185,190]

Solvent	Solute	NDP	$\rho_{n,1}^*$ range	T range (K)	k_{12}	B_{12}	AARD (%)	Data source
	ethyl benzoate	15	0.363-0.565	313.16-333.16	-0.27764	-0.34732	2.47	[182]
	ethylbenzene	15	0.363-0.565	313.15-333.15	0.09391	0.40509	1.54	[173]
	2-ethyltoluene	15	0.363-0.541	313.00-333.00	0.36063	2.22456	2.92	[213]
	3-ethyltoluene	15	0.363-0.541	313.00-333.00	0.36568	2.22371	3.35	[213]
	4-ethyltoluene	15	0.363-0.541	313.00-333.00	0.23924	1.27471	2.45	[213]
	eugenol	15	0.363-0.565	313.16-333.16	-0.11487	0.08537	2.41	[182]
	ferrocene	107	0.170-0.589	308.15-333.15	0.05896	0.42455	3.45	[208,214]
	2-fluoroanisole	15	0.363-0.565	313.16-333.16	0.20150	1.31776	1.36	[164]
	fluorobenzene	15	0.363-0.541	313.00-333.00	0.10752	0.82282	3.43	[183]
	3-fluorophenol	4	0.472-0.527	313.15-313.15	-0.39353	-0.41416	0.99	[196]
	geraniol	4	0.472-0.527	313.15-313.15	-0.38600	-0.42141	0.34	[196]
	n-heptane	5	0.442-0.497	299.15-308.15	-0.60907	-0.88892	1.34	[204]
	2-heptanone	11	0.386-0.490	314.45-314.45	-0.17692	-0.49695	1.69	[163]
	4-heptanone	9	0.376-0.479	313.45-313.45	0.05115	-0.21195	0.46	[163]
	hexachlorobenzene	14	0.244-0.545	308.00-328.00	0.75931	16.95813	4.59	[215]
	1-hexadecene	11	0.254-0.550	313.15-373.15	0.57283	5.75186	9.57	[216]
	1,1,1,5,5,5-hexafluoroacetylacetone	15	0.342-0.530	308.15-318.15	0.15376	1.09740	3.96	[201]
	n-hexane	5	0.442-0.497	299.15-308.15	-0.20484	-0.52061	2.17	[204]
	ibuprofen	99	0.260-0.588	308.00-353.15	0.19035	0.70041	2.87	[38]
	iodobenzene	20	0.363-0.565	313.00-333.00	0.28569	1.58290	2.42	[183,184]
	isobutylbenzene	15	0.363-0.565	313.00-333.00	0.05132	0.31174	1.92	[13]
	D-limonene	15	0.267-0.509	313.15-333.15	0.62916	7.91043	1.94	[199]
	linalool	15	0.267-0.509	313.15-333.15	0.24526	1.09629	3.38	[171]
	linoleic acid	71	0.331-0.564	308.15-343.15	0.46216	3.15391	2.91	[168]
	linoleic acid methyl ester	20	0.443-0.561	308.20-328.20	0.29058	1.13214	1.31	[152,217]

Solvent	Solute	NDP	$\rho_{n,1}^*$ range	T range (K)	k_{12}	B_{12}	AARD (%)	Data source
	α-linolenic acid	56	0.324-0.563	308.15-343.15	0.39721	2.11541	2.07	[212]
	γ-linolenic acid	142	0.272-0.551	308.15-343.15	0.42537	2.18606	3.32	[218]
	γ-linolenic acid ethyl ester	41	0.203-0.480	313.21-343.15	0.77314	19.15240	4.6	[218]
	γ-linolenic acid methyl ester	52	0.175-0.561	313.15-343.15	0.35971	1.59980	4.75	[217,218]
	L-menthone	23	0.388-0.563	308.15-338.15	-0.21905	-0.29426	3.01	[197]
	methanol	10	0.350-0.513	313.21-313.21	0.61329	10.32000	2.02	[179]
	2-methylanisole	15	0.363-0.565	313.00-333.00	0.21276	1.30032	1.99	[207]
	4-methylanisole	15	0.363-0.565	313.00-333.00	-0.01878	0.36370	1.83	[207]
	3-methylbutylbenzene	15	0.363-0.565	313.00-333.00	0.12978	0.58633	2.23	[13]
	1-methylnaphthalene	11	0.254-0.550	313.15-373.15	0.42515	3.26990	8.65	[216]
	monoolein	11	0.380-0.531	313.21-313.21	0.36267	1.66650	1.07	[211]
	myristic acid ethyl ester	16	0.361-0.514	308.00-318.00	0.00538	0.07254	1.97	[188,189]
	myristoleic acid	42	0.273-0.531	313.21-343.15	0.63627	7.83845	3.35	[219]
	myristoleic acid methyl ester	81	0.134-0.531	313.15-343.15	0.71596	13.43995	6.83	[219,220]
	naphthalene	114	0.133-0.673	288.25-333.15	0.38811	2.00230	9.13	[158,192,209,214,221]
	1-naphthol	11	0.319-0.495	308.00-318.00	-0.09672	-0.11272	0.54	[167]
	2-naphthol	16	0.197-0.487	308.00-328.00	-0.13286	-0.16679	0.86	[167]
	2-nitroanisole	15	0.363-0.565	313.00-333.00	0.19354	1.06652	1.43	[187]
	nitrobenzene	23	0.363-0.565	313.00-333.00	0.14108	0.76353	1.79	[166,184]
	3-nitrotoluene	15	0.363-0.565	313.00-333.00	0.13683	0.62982	2.18	[207]
	n-nonane	5	0.442-0.497	299.15-308.15	-0.56541	-0.83493	1.67	[204]
	2-nonanone	10	0.362-0.466	314.45-314.45	0.04378	-0.17274	2.29	[163]
	5-nonanone	12	0.362-0.490	314.45-314.45	-0.09396	-0.38512	1.02	[163]
	n-octane	5	0.442-0.497	299.15-308.15	-0.68972	-0.90856	1.91	[204]
	oleic acid	19	0.346-0.550	313.21-313.21	0.45886	3.09689	1.75	[211]

Solvent	Solute	NDP	$\rho_{n,1}^{*}$ range	T range (K)	k_{12}	B_{12}	AARD (%)	Data source
	oleic acid ethyl ester	5	0.228-0.413	313.21-313.21	0.77251	18.42052	2.96	[211]
	oleic acid methyl ester	21	0.168-0.481	313.00-313.21	0.84556	42.88455	4.86	[211,217,222]
	palladium(II) acetylacetonate	125	0.337-0.589	308.15-343.15	0.16111	1.11905	2	[200]
	palmitic acid ethyl ester	17	0.361-0.514	308.00-318.00	0.03578	0.15239	0.62	[155]
	n-pentane	5	0.442-0.497	299.15-308.15	-0.57435	-0.89519	2.15	[204]
	2-pentanone	23	0.335-0.547	308.15-314.50	0.13964	0.66390	1.83	[159]
	3-pentanone	46	0.355-0.575	308.15-328.15	0.16380	0.69224	1.92	[159,203]
	2,4-dimethyl-3-pentanone	8	0.367-0.491	314.15-314.15	-0.15013	-0.03387	2.21	[163]
	2,2,4,4-tetramethyl-3-pentanone	9	0.376-0.479	313.45-313.45	-0.07791	-0.31737	0.7	[203]
	n-pentylbenzene	31	0.363-0.577	308.00-398.00	0.12451	0.55848	2.09	[186]
	phenanthrene	25	0.303-0.555	303.15-333.15	0.68318	8.51016	3.61	[158,162,215]
	phenol	109	0.213-0.564	308.00-328.15	0.18750	1.03686	2.52	[160,190,194,195]
	phenylacetic acid	16	0.361-0.514	308.15-318.15	0.04211	0.30396	1.42	[223]
	phenylacetylene	15	0.363-0.565	313.16-333.16	0.19979	1.12486	1.13	[206]
	phenylbutazone	78	0.268-0.588	308.15-343.15	0.40882	2.13045	2.74	[40]
	1-phenyldodecane	15	0.363-0.565	313.00-333.00	0.17453	0.78508	1.93	[186]
	1-phenylethanol	15	0.363-0.565	313.16-333.16	0.15686	0.92580	1.32	[224]
	2-phenylethanol	15	0.363-0.565	313.16-333.16	0.19175	1.14300	1.42	[224]
	2-phenylethyl acetate	15	0.363-0.565	313.16-333.16	0.27775	1.67787	1.38	[34]
	1-phenylhexane	15	0.363-0.565	313.00-333.00	0.18119	0.79322	1.89	[186]
	phenylmethanol	15	0.363-0.565	313.16-333.16	0.07781	0.56424	1.55	[224]
	1-phenyloctane	15	0.363-0.565	313.00-333.00	0.29436	1.42529	2.08	[186]
	3-phenylpropyl acetate	15	0.363-0.565	313.16-333.16	0.44389	3.60577	1.34	[34]
	α-pinene	30	0.267-0.541	313.15-333.15	-0.02249	0.07574	3.04	[225,226]

Solvent	Solute	NDP	$\rho^*_{n,1}$ range	T range (K)	k_{12}	B_{12}	AARD (%)	Data source
	β-pinene	15	0.267-0.509	313.15-333.15	0.30695	1.36877	2.96	[225]
	1-propanol	17	0.350-0.480	313.21-313.21	0.72212	19.63428	2.84	[179]
	2-phenyl-1-propanol	15	0.363-0.565	313.16-333.16	0.20514	1.25963	1.51	[224]
	2-propanol	18	0.350-0.488	313.21-313.21	0.76258	27.26173	2.2	[179]
	3-phenyl-1-propanol	15	0.363-0.565	313.16-333.16	-0.01099	0.22401	1.6	[224]
	i-propylbenzene	36	0.303-0.565	313.15-333.15	0.18942	0.83024	2.24	[158,173,185,227]
	n-propylbenzene	60	0.216-0.565	308.15-333.15	0.57874	6.40360	4.5	[172,173,185,227]
	pyrene	21	0.233-0.563	303.15-333.15	0.00977	0.10193	2.03	[158,167]
	squalene	5	0.442-0.490	314.45-314.45	-0.21802	-0.42379	1.74	[163]
	stearic acid	4	0.447-0.479	313.50-313.50	0.12914	-0.16980	0.38	[163]
	stearic acid ethyl ester	17	0.361-0.514	308.00-318.00	0.09804	0.21940	0.67	[170]
	styrene	15	0.363-0.541	313.16-333.16	0.20429	1.23722	3.85	[165]
	n-tetradecane	5	0.442-0.497	299.15-308.15	-0.59534	-0.76334	3.23	[204]
	tetrahydrofuran	15	0.117-0.482	313.15-333.15	0.83293	57.62331	8.78	[205]
	thenoyltrifluoroacetone	15	0.342-0.535	308.15-318.15	0.02762	0.54036	3.15	[201]
	α-tocopherol	82	0.372-0.564	308.15-333.15	0.36960	1.31453	0.88	[193–195]
	toluene	41	0.129-0.565	306.12-333.15	0.23069	1.22904	9.72	[173,176,190]
	triarachidonin	27	0.377-0.550	313.21-313.21	0.61908	4.16419	0.71	[228]
	trierucin	101	0.342-0.563	308.15-323.15	0.80342	17.06793	1.88	[228]
	trifluoroacetylacetone	15	0.349-0.531	308.15-318.15	0.08009	0.47331	1.59	[201]
	1,3,5-trimethylbenzene	34	0.351-0.565	303.15-333.16	0.37061	2.42310	3.82	[158,165,172,184]
	trinervonin	38	0.359-0.563	308.15-323.15	0.72743	8.27233	1.85	[228]
	triolein	14	0.312-0.574	298.10-313.21	0.59231	2.70604	2.04	[180,228]
	ubiquinone CoQ10	80	0.372-0.563	308.15-333.15	0.44028	1.64757	1.57	[195,229]
	n-undecane	5	0.442-0.497	299.15-308.15	-0.37897	-0.71665	2.04	[204]
	6-undecanone	13	0.362-0.490	314.45-314.45	-0.01004	-0.24598	2.16	[163]

Solvent	Solute	NDP	$\rho^*_{n,1}$ range	T range (K)	k_{12}	B_{12}	AARD (%)	Data source
	vanillin	15	0.361-0.514	308.15-318.15	0.05824	0.43669	1.7	[223]
	vitamin K_1	17	0.380-0.550	313.20-313.20	0.40163	1.10971	2.33	[202,220]
	vitamin K_3	22	0.288-0.550	313.15-313.15	0.38920	1.71033	2.53	[152,195,220]
	water	24	0.481-0.625	283.15-308.15	-1.32022	-1.14870	9.97	[230]
	5-*tert*-butyl-*m*-xylene	31	0.362-0.577	308.16-398.16	0.05177	0.28237	2.12	[206]
	m-xylene	12	0.111-0.550	313.15-373.15	0.70874	19.04885	14.95	[216]
	p-xylene	7	0.421-0.531	313.15-323.15	0.06005	0.26185	3.04	[184,185]

References

1. Valderrama, J.O.; Rojas, R.E. Critical Properties of Ionic Liquids. Revisited. *Ind. Eng. Chem. Res.* **2009**, *48*, 6890–6900.
2. Joback, K.G.; Reid, R.C. A Unified Approach to Physical Property Estimation Using Multivariate Statistical Techniques, Massachusetts Institute of Technology: Cambridge, MA, 1984.
3. Joback, K.G.; Reid, R.C. Estimation of Pure-Component Properties from Group-Contributions. *Chem. Eng. Commun.* **1987**, *57*, 233–243.
4. Poling, B.E.; Prausnitz, J.M.; O'Connell, J.P. *The Properties of Gases and Liquids*; Company, M.-H.B., Ed.; Fifth ed.; The McGraw-Hill Companies, Inc: New York, 2001; ISBN 0071499997.
5. Somayajulu, G.R. Estimation Procedures for Critical Constants. *J. Chem. Eng. Data* **1989**, *34*, 106–120.
6. Yaws, C.L. *Thermophysical Properties of Chemicals and Hydrocarbons*; William Andrew Inc.: New York, 2008;
7. Reid, R.C.; Prausnitz, J.M.; Poling, B.E. *The Properties of Gases and Liquids*; Company, M.-H.B., Ed.; Fourth ed.; McGraw-Hill International Editions: New York, 1987;
8. Nikitin, E.D.; Popov, A.P. Critical Point Measurement of Ferrocene and Some of Its Derivatives. *Fluid Phase Equilib.* **2012**, *324*, 13–16.
9. Magalhães, A.L.; Da Silva, F.A.; Silva, C.M. Free-Volume Model for the Diffusion Coefficients of Solutes at Infinite Dilution in Supercritical CO_2 and Liquid H_2O. *J. Supercrit. Fluids* **2013**, *74*, 89–104.
10. Klincewicz, K.M.; Reid, R.C. Estimation of Critical Properties with Group Contribution Methods. *AIChE J.* **1984**, *30*, 137–142.
11. Green, D.W.; Perry, R.H. *Perry's Chemical Engineers' Handbook*; 8th ed.; McGraw-Hill Professional: New York, 2008;
12. Wen, X.; Qiang, Y. A New Group Contribution Method for Estimating Critical Properties of Organic Compounds. *Ind. Eng. Chem. Res.* **2001**, *40*, 6245–6250.
13. Pizarro, C.; Suárez-Iglesias, O.; Medina, I.; Bueno, J.L. Diffusion Coefficients of Isobutylbenzene, Sec-Butylbenzene, and 3-Methylbutylbenzene in Supercritical Carbon Dioxide. *J. Chem. Eng. Data* **2013**, *58*, 2001–2007.
14. AIChE Design Institute for Physical Properties (DIPPR) Available online: http://dippr.byu.edu/ (accessed on 22 August 2020).
15. Liu, H.; Ruckenstein, E. A Predictive Equation for the Tracer Diffusion of Various Solutes in Gases, Supercritical Fluids, and Liquids. *Ind. Eng. Chem. Res.* **1997**, *36*, 5488–5500.
16. Constantinou, L.; Gani, R. New Group Contribution Method for Estimating Properties of Pure Compounds. *AIChE J.* **1994**, *40*, 1697–1710.
17. Molecular Design LAB. (Dept. of Chemical Engineering) Korea Thermophysical Properties Data Bank (KDB) Available online: http://www.cheric.org/research/kdb/hcprop/cmpsrch.php (accessed on 22 August 2020).
18. Zêzere, B.; Magalhães, A.L.; Portugal, I.; Silva, C.M. Diffusion Coefficients of Eucalyptol at Infinite Dilution in Compressed Liquid Ethanol and in Supercritical CO_2/Ethanol Mixtures. *J. Supercrit. Fluids* **2018**, *133*, 297–308.
19. Leite, J.; Magalhães, A.L.; Valente, A.A.; Silva, C.M. Measurement and Modelling of Tracer Diffusivities of Gallic Acid in Liquid Ethanol and in Supercritical CO_2 Modified with Ethanol. *J. Supercrit. Fluids* **2018**, *131*, 130–139.
20. Magalhães, A.L.; Da Silva, F.A.; Silva, C.M. New Tracer Diffusion Correlation for Real Systems over Wide Ranges of Temperature and Density. *Chem. Eng. J.* **2011**, *166*, 49–72.
21. Catchpole, O.J.; Von Kamp, J.C. Phase Equilibrium for the Extraction of Squalene from Shark Liver Oil Using Supercritical Carbon Dioxide. *Ind. Eng. Chem. Res.* **1997**, *36*, 3762–3768.
22. Man, C.W. Limiting Mutual Diffusion of Nonassociated Aromatic Solutes, The Hong Kong Polytechnic University: Hong Kong, 2001.
23. Tyn, M.T.; Calus, W.F. Temperature and Concentration Dependence of Mutual Diffusion Coefficients of Some Binary Liquid Systems. *J. Chem. Eng. Data* **1975**, *20*, 310–316.
24. Cooper, E. Diffusion Coefficients at Infinite Dilution in Alcohol Solvents at Temperatures to 348 K and Pressures to 17 MPa, University of Ottawa: Canada, 1992.
25. Wong, C.-F.; Hayduk, W. Molecular Diffusivities for Propene in 1-Butanol, Chlorobenzene, Ethylene Glycol, and n-Octane at Elevated Pressures. *J. Chem. Eng. Data* **1990**, *35*, 323–328.
26. Wong, C.-F. Diffusion Coefficients of Dissolved Gases in Liquids, University of Ottawa: Canada, 1989.
27. Kopner, A.; Hamm, A.; Ellert, J.; Feist, R.; Schneider, G.M. Determination of Binary Diffusion Coefficients in Supercritical Chlorotrifluoromethane and Sulfurhexafluoride with Supercritical Fluid Chromatography (SFC). *Chem. Eng. Sci.* **1987**, *42*, 2213–2218.

28. Han, P.; Bartels, D.M. Temperature Dependence of Oxygen Diffusion in H2O and D2O. *J. Phys. Chem.* **1996**, *100*, 5597–5602.
29. Okubo, Y.; Kong, C.Y.; Sakabe, J.; Funazukuri, T. Measurements of Infinite Dilution Binary Diffusion Coefficients of Acetylferrocene and 1,1′-Diacetylferrocene in Supercritical Carbon Dioxide and in Liquid Organic Solvents. *J. Supercrit. Fluids* **2021**, *177*, 105321.
30. Kong, C.Y.; Watanabe, K.; Funazukuri, T. Determination and Correlation of Infinite Dilution Binary Diffusion Coefficients for Aluminum Acetylacetonate in Supercritical and Liquid Fluids. *Fluid Phase Equilib.* **2015**, *420*, 83–88.
31. Sun, C.K.J.; Chen, S.H. Tracer Diffusion in Dense Ethanol: A Generalized Correlation for Nonpolar and Hydrogen-Bonded Solvents. *AIChE J.* **1986**, *32*, 1367–1371.
32. Tominaga, T.; Matsumoto, S. Diffusion of Polar and Nonpolar Molecules in Water and Ethanol. *Bull. Chem. Soc. Jpn.* **1990**, *63*, 533–537.
33. Lin, I.-H.; Tan, C.-S. Diffusion of Benzonitrile in CO_2 - Expanded Ethanol. *J. Chem. Eng. Data* **2008**, *53*, 1886–1891.
34. Suárez-Iglesias, O.; Medina, I.; Pizarro, C.; Bueno, J.L. Diffusion of Benzyl Acetate, 2-Phenylethyl Acetate, 3-Phenylpropyl Acetate, and Dibenzyl Ether in Mixtures of Carbon Dioxide and Ethanol. *Ind. Eng. Chem. Res.* **2007**, *46*, 3810–3819.
35. Zêzere, B.; Cordeiro, J.; Leite, J.; Magalhães, A.L.; Portugal, I.; Silva, C.M. Diffusivities of Metal Acetylacetonates in Liquid Ethanol and Comparison with the Transport Behavior in Supercritical Systems. *J. Supercrit. Fluids* **2019**, *143*, 259–267.
36. Kong, C.Y.; Watanabe, K.; Funazukuri, T. Measurement and Correlation of the Diffusion Coefficients of Chromium(III) Acetylacetonate at Infinite Dilution in Supercritical Carbon Dioxide and in Liquid Ethanol. *J. Chem. Thermodyn.* **2017**, *105*, 86–93.
37. Funazukuri, T.; Yamasaki, T.; Taguchi, M.; Kong, C.Y. Measurement of Binary Diffusion Coefficient and Solubility Estimation for Dyes in Supercritical Carbon Dioxide by CIR Method. *Fluid Phase Equilib.* **2015**, *420*, 7–13.
38. Kong, C.Y.; Sugiura, K.; Natsume, S.; Sakabe, J.; Funazukuri, T.; Miyake, K.; Okajima, I.; Badhulika, S.; Sako, T. Measurements and Correlation of Diffusion Coefficients of Ibuprofen in Both Liquid and Supercritical Fluids. *J. Supercrit. Fluids* **2020**, *159*, 104776.
39. Snijder, E.D.; te Riele, M.J.M.; Versteeg, G.F.; van Swaaij, W.P.M. Diffusion Coefficients of CO, CO2, N2O, and N2 in Ethanol and Toluene. *J. Chem. Eng. Data* **1995**, *40*, 37–39.
40. Kong, C.Y.; Watanabe, K.; Funazukuri, T. Diffusion Coefficients of Phenylbutazone in Supercritical CO_2 and in Ethanol. *J. Chromatogr. A* **2013**, *1279*, 92–97.
41. Zêzere, B.; Iglésias, J.; Portugal, I.; Gomes, J.R.B.; Silva, C.M. Diffusion of Quercetin in Compressed Liquid Ethyl Acetate and Ethanol. *J. Mol. Liq.* **2020**, 114714.
42. Pratt, K.C.; Wakeham, W.A. The Mutual Diffusion Coefficient of Ethanol-Water Mixtures: Determination by a Rapid, New Method. *Proc. R. Soc. London A* **1974**, *336*, 393–406.
43. Zêzere, B.; Silva, J.M.; Portugal, I.; Gomes, J.R.B.; Silva, C.M. Measurement of Astaxanthin and Squalene Diffusivities in Compressed Liquid Ethyl Acetate by Taylor-Aris Dispersion Method. *Sep. Purif. Technol.* **2020**, *234*, 116046.
44. Heintz, A.; Ludwig, R.; Schmidt, E. Limiting Diffusion Coefficients of Ionic Liquids in Water and Methanol: A Combined Experimental and Molecular Dynamics Study. *Phys. Chem. Chem. Phys.* **2011**, *13*, 3268–3273.
45. Sarraute, S.; Gomes, M.F.C.; Pádua, A.A.H. Diffusion Coefficients of 1-Alkyl-3-Methylimidazolium Ionic Liquids in Water, Methanol, and Acetonitrile at Infinite Dilution. *J. Chem. Eng. Data* **2009**, *54*, 2389–2394.
46. Hurle, R.L.; Woolf, L.A. Tracer Diffusion in Methanol and Acetonitrile under Pressure. *J. Chem. Soc. Faraday Trans. 1* **1982**, *78*, 2921–2928.
47. Sun, C.K.J.; Chen, S.H. Tracer Diffusion in Dense Methanol and 2-Propanol up to Supercritical Region: Understanding of Solvent Molecular Association and Development of an Empirical Correlation. *Ind. Eng. Chem. Res.* **1987**, *26*, 815–819.
48. Liu; Takemura, F.; Yabe, A. Solubility and Diffusivity of Carbon Monoxide in Liquid Methanol. *J. Chem. Eng. Data* **1996**, *41*, 589–592.
49. Lin, I.-H.; Tan, C.-S. Measurement of Diffusion Coefficients of P-Chloronitrobenzene in CO2-Expanded Methanol. *J. Supercrit. Fluids* **2008**, *46*, 112–117.
50. Funazukuri, T.; Sugihara, T.; Yui, K.; Ishii, T.; Taguchi, M. Measurement of Infinite Dilution Diffusion Coefficients of Vitamin K_3 in CO_2 Expanded Methanol. *J. Supercrit. Fluids* **2016**, *108*, 19–25.
51. Lee, Y.E.; Li, F.Y. Binary Diffusion Coefficients of the Methanol Water System in the Temperature Range 30-40 ^{o}C. *J. Chem. Eng. Data* **1991**, *36*, 240–243.

52. Pratt, K.C.; Wakeham, W.A. The Mutual Diffusion Coefficient for Binary Mixtures of Water and the Isomers of Propanol. *Proc. R. Soc. London A* **1975**, *342*, 401–419.
53. Wong, C.-L.; Soriano, A.N.; Li, M.-H. Infinite Dilution Diffusion Coefficients of [Bmim]-Based Ionic Liquids in Water and Its Molar Conductivities. *J. Taiwan Inst. Chem. Eng.* **2009**, *40*, 77–83.
54. Wong, C.-L.; Soriano, A.N.; Li, M.-H. Diffusion Coefficients and Molar Conductivities in Aqueous Solutions of 1-Ethyl-3-Methylimidazolium-Based Ionic Liquids. *Fluid Phase Equilib.* **2008**, *271*, 43–52.
55. Heintz, A.; Lehmann, J.K.; Schmidt, E.; Wandschneider, A. Diffusion Coefficients of Imidazolium Based Ionic Liquids in Aqueous Solutions. *J. Solution Chem.* **2009**, *38*, 1079–1083.
56. Gary-Bobo, C.; Weber, H.W. Diffusion of Alcohols and Amides in Water from 4 to 37.Deg. *J. Phys. Chem.* **1969**, *73*, 1155–1156.
57. Umecky, T.; Kuga, T.; Funazukuri, T. Infinite Dilution Binary Diffusion Coefficients of Several α-Amino Acids in Water over a Temperature Range from (293.2 to 333.2) K with the Taylor Dispersion Technique. *J. Chem. Eng. Data* **2006**, *51*, 1705–1710.
58. Umecky, T.; Omori, S.; Kuga, T.; Funazukuri, T. Effects of Hydroxyl Groups on Binary Diffusion Coefficients of α-Amino Acids in Dilute Aqueous Solutions. *Fluid Phase Equilib.* **2008**, *264*, 18–22.
59. Frank, M.J.W.; Kuipers, J.A.M.; van Swaaij, W.P.M. Diffusion Coefficients and Viscosities of CO2+H2O, CO2+CH3OH, NH3+H2O, and NH3+CH3OH Liquid Mixtures. *J. Chem. Eng. Data* **1996**, *41*, 297–302.
60. Mogi, N.; Sugai, E.; Fuse, Y.; Funazukuri, T. Infinite Dilution Binary Diffusion Coefficients for Six Sugars at 0.1 MPa and Temperatures from (273.2 to 353.2) K. *J. Chem. Eng. Data* **2007**, *52*, 40–43.
61. Maharajh, D. Solubility and Diffusion of Gases in Water, Simon Fraser University: British Columbia, 1975.
62. Witherspoon, P.A.; Bonoli, L. Correlation of Diffusion Coefficients for Paraffin, Aromatic, and Cycloparaffin Hydrocarbons in Water. *Ind. Eng. Chem. Fundam.* **1969**, *8*, 589–591.
63. Bonoli, L.; Witherspoon, P.A. Diffusion of Aromatic and Cycloparaffin Hydrocarbons in Water from 2 to 60.Deg. *J. Phys. Chem.* **1968**, *72*, 2532–2534.
64. Tominaga, T.; Yamamoto, S.; Takanaka, J.-I. Limiting Interdiffusion Coefficients of Benzene, Toluene, Ethylbenzene and Hexafluorobenzene in Water from 298 to 368 K. *J. Chem. Soc. Faraday Trans. 1 Phys. Chem. Condens. Phases* **1984**, *80*, 941.
65. Tominaga, T.; Matsumoto, S.; Ishii, T. Limiting Interdiffusion of Some Aromatic Hydrocarbons in Water from 265 to 433 K. *J. Phys. Chem.* **1986**, *90*, 139–143.
66. Delgado, J.M.P.Q. Molecular Diffusion Coefficients of Organic Compounds in Water at Different Temperatures. *J. Phase Equilibria Diffus.* **2007**, *28*, 427–432.
67. Mutoru, J.W.; Leahy-Dios, A.; Firoozabadi, A. Modeling Infinite Dilution and Fickian Diffusion Coefficients of Carbon Dioxide in Water. *AIChE J.* **2011**, *57*, 1617–1627.
68. Jähne, B.; Heinz, G.; Dietrich, W. Measurement of the Diffusion Coefficients of Sparingly Soluble Gases in Water. *J. Geophys. Res.* **1987**, *92*, 10767–10776.
69. Riele, M.J.M.T.; Snijder, E.D.; van Swaaij, W.P.M. Diffusion Coefficients at Infinite Dilution in Water and in N-Methylpyrrolidone. *J. Chem. Eng. Data* **1995**, *40*, 34–36.
70. Li, S.F.Y.; Ong, H.M. Infinite Dilution Diffusion Coefficients of Several Alcohols in Water. *J. Chem. Eng. Data* **1990**, *35*, 136–137.
71. Tominaga, T.; Matsumoto, S. Limiting Interdiffusion Coefficients of Some Hydroxylic Compounds in Water from 265 to 433 K. *J. Chem. Eng. Data* **1990**, *35*, 45–47.
72. Funazukuri, T.; Nishio, M. Infinite Dilution Binary Diffusion Coefficients of C5-Monoalcohols in Water in the Temperature Range from 273.2 K to 353.2 K at 0.1 MPa. *J. Chem. Eng. Data* **1999**, *44*, 73–76.
73. Price, W.E.; Trickett, K.A.; Harris, K.R. Association of Caffeine in Aqueous Solution. Effects on Caffeine Intradiffusion. *J. Chem. Soc. Faraday Trans. 1* **1989**, *85*, 3281–3288.
74. Price, W.E. Tracer Caffeine Diffusion in Aqueous Solutions at 298 K. The Effect of Caffeine Self-Association. *J. Chem. Soc. Faraday Trans. 1* **1989**, *85*, 415–419.
75. Niesner, R.; Heintz, A. Diffusion Coefficients of Aromatics in Aqueous Solution. *J. Chem. Eng. Data* **2000**, *45*, 1121–1124.
76. Vivian, J.E.; King, C.J. Diffusivities of Slightly Soluble Gases in Water. *AIChE J.* **1964**, *10*, 220–221.
77. Ng, W.Y.; Walkley, J. Diffusion of Gases in Liquids: The Constant Size Bubble Method. *Can. J. Chem.* **1969**, *47*, 1075–1077.
78. Maharajh, D.M.; Walkley, D.J. The Temperature Dependence of the Diffusion Coefficients of Ar, CO_2, CH_4, CH_3Cl, CH_3Br, and $CHCl_2F$ in Water. *Can. J. Chem.* **1973**, *51*, 944–952.
79. Pratt, K.C.; Slater, D.H.; Wakeham, W.A. A Rapid Method for the Determination of Diffusion Coefficients of Gases in Liquids. *Chem. Eng. Sci.* **1973**, *28*, 1901–1903.
80. Bodnar, L.H.; Himmelblau, D.M. Continuous Measurement of Diffusion Coefficients of Gases in Liquids Using Glass Scintillators. *Int. J. Appl. Radiat. Isot.* **1962**, *13*, 1–6.

81. Woods, D.R. Mass Transfer Between a Liquid Jet and a Countercurrent Gas Stream, University of Wisconsin-Madison: Wisconsin, 1961.
82. Ferrell, R.T.; Himmelblau, D.M. Diffusion Coefficients of Nitrogen and Oxygen in Water. *J. Chem. Eng. Data* **1967**, *12*, 111–115.
83. Thomas, W.J.; Adams, M.J. Measurement of the Diffusion Coefficients of Carbon Dioxide and Nitrous Oxide in Water and Aqueous Solutions of Glycerol. *Trans. Faraday Soc.* **1965**, *61*, 668–673.
84. Tham, M.J.; Bhatia, K.K.; Gubbins, K.F. Steady-State Method for Studying Diffusion of Gases in Liquids. *Chem. Eng. Sci.* **1967**, *22*, 309–311.
85. Sovová, H.; Procházka, J. A New Method of Measurement of Diffusivities of Gases in Liquids. *Chem. Eng. Sci.* **1976**, *31*, 1091–1097.
86. Himmelblau, D.M. Diffusion of Dissolved Gases in Liquids. *Chem. Rev.* **1964**, *64*, 527–550.
87. Peaceman, D.W. Liquid-Side Resistance in Gas Absorption with and without Chemical Reaction, Massachusetts Institute of Technology: Massachusetts, 1952.
88. Ringbom, A. Über Die Bestimmung Der Diffusionskoeffizienten von Gasen in Flüssigkeiten. *Zeitschrift für Anorg. und Allg. Chemie* **1938**, *238*, 94–102.
89. Norman, W.S.; Sammak, F.Y.Y. Gas Absorption in a Packed Column - Part I: The Effect of Liquid Viscosity on the Mass Transfer Coefficient. *Trans. Inst. Chem. Eng.* **1963**, *41*, 109–116.
90. Onda, K.; Okamoto, T.; Yamaji, Y. Measurement of the Diffusivities of CO_2 in Liquids by Liquid Jets. *Kagaku Kogaku* **1960**, *24*, 918–925.
91. Nijsing, R.A.T.O.; Hendriksz, R.H.; Kramers, H. Absorption of CO_2 in Jets and Falling Films of Electrolyte Solutions, with and without Chemical Reaction. *Chem. Eng. Sci.* **1959**, *10*, 88–104.
92. Hüfner, G. Ueber Die Bestimmung Der Diffusions Coefficienten Einiger Gase Für Wasser. *Ann. Phys.* **1896**, *296*, 134–168.
93. Stefan, J. Über Die Diffusion Der Kohlensäure Durch Wasser Und Alkohol. *Sitzungsberichte der Kais. Akad. der Wissenschaften, Math. Klasse, II Abtheilung* **1878**, *77*, 371–409.
94. Tammann, G.; Jessen, V. Über Die Diffusionskoeffizienten von Gasen in Wasser Und Ihre Temperaturabhängigkeit. *Zeitschrift für Anorg. und Allg. Chemie* **1929**, *179*, 125–144.
95. Tamimi, A.; Rinker, E.B.; Sandall, O.C. Diffusion Coefficients for Hydrogen Sulfide, Carbon Dioxide, and Nitrous Oxide in Water over the Temperature Range 293-368 K. *J. Chem. Eng. Data* **1994**, *39*, 330–332.
96. Versteeg, G.F.; Van Swaaij, W.P.M. Solubility and Diffusivity of Acid Gases (Carbon Dioxide, Nitrous Oxide) in Aqueous Alkanolamine Solutions. *J. Chem. Eng. Data* **1988**, *33*, 29–34.
97. Cadogan, S.P.; Maitland, G.C.; Trusler, J.P.M. Diffusion Coefficients of CO_2 and N_2 in Water at Temperatures between 298.15 K and 423.15 K at Pressures up to 45 MPa. *J. Chem. Eng. Data* **2014**, *59*, 519–525.
98. Mazarei, A.F.; Sandall, O.C. Diffusion Coefficients for Helium, Hydrogen, and Carbon Dioxide in Water at 25°C. *AIChE J.* **1980**, *26*, 154–157.
99. Ribeiro, A.C.F.; Valente, A.J.M.; Santos, C.I.A. V; Prazeres, P.M.R.A.; Lobo, V.M.M.; Burrows, H.D.; Esteso, M.A.; Cabral, A.M.T.D.P. V; Veiga, F.J.B. Binary Mutual Diffusion Coefficients of Aqueous Solutions of α-Cyclodextrin, 2-Hydroxypropyl-α-Cyclodextrin, and 2-Hydroxypropyl-β-Cyclodextrin at Temperatures from (298.15 to 312.15) K. *J. Chem. Eng. Data* **2007**, *52*, 586–590.
100. Ribeiro, A.C.F.; Leaist, D.G.; Esteso, M.A.; Lobo, V.M.M.; Valente, A.J.M.; Santos, C.I.A. V; Cabral, A.M.T.D.P. V; Veiga, F.J.B. Binary Mutual Diffusion Coefficients of Aqueous Solutions of β-Cyclodextrin at Temperatures from 298.15 to 312.15 K. *J. Chem. Eng. Data* **2006**, *51*, 1368–1371.
101. Snijder, E.D.; te Riele, M.J.M.; Versteeg, G.F.; van Swaaij, W.P.M. Diffusion Coefficients of Several Aqueous Alkanolamine Solutions. *J. Chem. Eng. Data* **1993**, *38*, 475–480.
102. Wang, M.H.; Soriano, A.N.; Caparanga, A.R.; Li, M.-H. Mutual Diffusion Coefficients of Aqueous Solutions of Some Glycols. *Fluid Phase Equilib.* **2009**, *285*, 44–49.
103. Secuianu, C.; Maitland, G.C.; Trusler, J.P.M.; Wakeham, W.A. Mutual Diffusion Coefficients of Aqueous KCl at High Pressures Measured by the Taylor Dispersion Method. *J. Chem. Eng. Data* **2011**, *56*, 4840–4848.
104. Kuo, C.-C.; Li, M.-H. Measurements of Mutual Diffusion Coefficients of Binary Systems: Aquous N,N-Diethylethanolamine, N,N-Dimethylethanolamine, Monoisopropanolamine, Piperazine, and Sulfolane Solutions, Chung Yuan Christian University : Jhongli, 2005, Vol. Master.
105. Chang, L.-C.; Lin, T.-I.; Li, M.-H. Mutual Diffusion Coefficients of Some Aqueous Alkanolamines Solutions. *J. Chem. Eng. Data* **2005**, *50*, 77–84.
106. Wang, M.H.; Soriano, A.N.; Caparanga, A.R.; Li, M.-H. Binary Mutual Diffusion Coefficient of Aqueous Solutions of Propylene Glycol and Dipropylene Glycol. *J. Taiwan Inst. Chem. Eng.* **2010**, *41*, 279–285.

107. Kuo, C.-C.; Li, M.H. Diffusion Coefficients of Aqueous N,N-Diethylethanolamine, N,N-Dimethylethanolamine, Monoisopropanolamine, Piperazine, and Sulfolane Solutions. *J. Chinese Inst. Chem. Eng.* **2006**, *37*, 341–353.
108. Witherspoon, P.A.; Saraf, D.N. Diffusion of Methane, Ethane, Propane and n-Butane in Water from 25 to 43°. *J. Phys. Chem.* **1965**, *69*, 3752–3755.
109. Ribeiro, A.C.F.; Ortona, O.; Simões, S.M.N.; Santos, C.I.A. V; Prazeres, P.M.R.A.; Valente, A.J.M.; Lobo, V.M.M.; Burrows, H.D. Binary Mutual Diffusion Coefficients of Aqueous Solutions of Sucrose, Lactose, Glucose, and Fructose in the Temperature Range from (298.15 to 328.15) K. *J. Chem. Eng. Data* **2006**, *51*, 1836–1840.
110. Yui, K.; Yamazaki, N.; Funazukuri, T. Infinite Dilution Binary Diffusion Coefficients for Compounds Derived from Biomass in Water at 0.1 MPa and Temperatures from (298.2 to 353.2) K. *J. Chem. Eng. Data* **2013**, *58*, 183–186.
111. Boerboom, A.J.; Kleyn, G. Diffusion Coefficients of Noble Gases in Water. *J. Chem. Phys.* **1969**, *50*, 1086–1088.
112. Haimour, N.; Sandall, O.C. Molecular Diffusivity of Hydrogen Sulfide in Water. *J. Chem. Eng. Data* **1984**, *29*, 20–22.
113. Derlacki, Z.J.; Easteal, A.J.; Edge, A.V.J.; Woolf, L.A.; Roksandic, Z. Diffusion Coefficients of Methanol and Water and the Mutual Diffusion Coefficient in Methanol-Water Solutions at 278 and 298 K. *J. Phys. Chem.* **1985**, *89*, 5318–5322.
114. Matthews, M.A.; Akgerman, A. Infinite Dilution Diffusion Coefficients of Methanol and 2-Propanol in Water. *J. Chem. Eng. Data* **1988**, *33*, 122–123.
115. Derks, P.W.J.; Hamborg, E.S.; Hogendoorn, J.A.; Niederer, J.P.M.; Versteeg, G.F. Densities, Viscosities, and Liquid Diffusivities in Aqueous Piperazine and Aqueous (Piperazine + N-Methyldiethanolamine) Solutions. *J. Chem. Eng. Data* **2008**, *53*, 1179–1185.
116. Hamborg, E.S.; Derks, P.W.J.; Kersten, S.R.A.; Niederer, J.P.M.; Versteeg, G.F. Diffusion Coefficients of N_2O in Aqueous Piperazine Solutions Using the Taylor Dispersion Technique from (293 to 333) K and (0.3 to 1.4) Mol·dm −3. *J. Chem. Eng. Data* **2008**, *53*, 1462–1466.
117. Wise, D.L.; Houghton, G. The Diffusion Coefficients of Ten Slightly Soluble Gases in Water at 10–60°C. *Chem. Eng. Sci.* **1966**, *21*, 999–1010.
118. Plugatyr, A.; Svishchev, I.M. Molecular Diffusivity of Phenol in Sub- and Supercritical Water: Application of the Split-Flow Taylor Dispersion Technique. *Jounal Phys. Chem. B* **2011**, *115*, 2555–2562.
119. Castillo, R.; Garza, C. Temperature Dependence of the Mutual Diffusion Coefficients in Aqueous Solutions of Alkali Metal Chlorides. *Int. J. Thermophys.* **1993**, *14*, 1145–1152.
120. Funazukuri, T.; Nishimoton; Wakao, N. Binary Diffusion Coefficients of Organic Compounds in Hexane, Dodecane, and Cyclohexane at 303.2-333.2 K and 16.0 MPa. *J. Chem. Eng. Data* **1994**, *39*, 911–915.
121. Chen, S.H.; Davis, H.T.; Evans, D.F. Tracer Diffusion in Polyatomic Liquids. II. *J. Chem. Phys.* **1981**, *75*, 2540–2544.
122. Sun, C.K.J.; Chen, S.H. Tracer Diffusion of Aromatic Hydrocarbons in Liquid Cyclohexane up to Its Critical Temperature. *AIChE J.* **1985**, *31*, 1510–1515.
123. Toriurmi, M.; Katooka, R.; Yui, K.; Funazukuri, T.; Kong, C.Y.; Kagei, S. Measurements of Binary Diffusion Coefficients for Metal Complexes in Organic Solvents by the Taylor Dispersion Method. *Fluid Phase Equilib.* **2010**, *297*, 62–66.
124. Chen, B.H.C.; Sun, C.K.J.; Chen, S.H. Hard Sphere Treatment of Binary Diffusion in Liquid at High Dilution up to the Critical Temperature. *J. Chem. Phys.* **1985**, *82*, 2052–2055.
125. Sun, C.K.J.; Chen, S.H. Tracer Diffusion of Aromatic Hydrocarbons in N-Hexane up to the Supercritical Region. *Chem. Eng. Sci.* **1985**, *40*, 2217–2224.
126. Chen, S.H.; Davis, H.T.; Evans, D.F. Tracer Diffusion in Polyatomic Liquids. III. *J. Chem. Phys.* **1982**, *77*, 2540–2544.
127. Chen, H.C.; Chen, S.H. Tracer Diffusion of Crown Ethers in N-Decane and n-Tetradecane: An Improved Correlation for Binary Systems Involving Normal Alkanes. *Ind. Eng. Chem. Fundam.* **1985**, *24*, 187–192.
128. Pollack, G.L.; Kennan, R.P.; Himm, J.F.; Stump, D.R. Diffusion of Xenon in Liquid Alkanes: Temperature Dependence Measurements with a New Method. Stokes--Einstein and Hard Sphere Theories. *J. Chem. Phys.* **1990**, *92*, 625–630.
129. Sun, C.K.J.; Chen, S.H. Diffusion of Benzene, Toluene, Naphthalene, and Phenanthrene in Supercritical Dense 2,3-Dimethylbutane. *AIChE J.* **1985**, *31*, 1904–1910.
130. Matthews, M.A.; Rodden, J.B.; Akgerman, A. High-Temperature Diffusion of Hydrogen, Carbon Monoxide, and Carbon Dioxide in Liquid n-Heptane, n-Dodecane, and n-Hexadecane. *J. Chem. Eng. Data* **1987**, *32*, 319–322.

131. Matthews, M.A.; Akgerman, A. Diffusion Coefficients for Binary Alkane Mixtures to 573 K and 3.5 MPa. *AIChE J.* **1987**, *33*, 881–885.
132. Rodden, J.B.; Erkey, C.; Akgerman, A. High-Temperature Diffusion, Viscosity, and Density Measurements in n-Eicosane. *J. Chem. Eng. Data* **1988**, *33*, 344–347.
133. Noel, J.M.; Erkey, C.; Bukur, D.B.; Akgerman, A. Infinite Dilution Mutual Diffusion Coefficients of 1-Octene and 1-Tetradecene in near-Critical Ethane and Propane. *J. Chem. Eng. Data* **1994**, *39*, 920–921.
134. Grushka, E.; Kikta, E.J. Diffusion in Liquids. II. Dependence of Diffusion Coefficients on Molecular Weight and on Temperature. *J. Am. Chem. Soc.* **1976**, *98*, 643–648.
135. Qian, R.Y.; Fan, Y.Q.; Shi, M.R.; Shi, J. Predictive Equation of Tracer Liquid Diffusion Coefficient from Viscosity. *Chinese J. Chem. Eng.* **1996**, *4*, 203–208.
136. Lo, H.Y. Diffusion Coefficients in Binary Liquid N-Alkane Systems. *J. Chem. Eng. Data* **1974**, *19*, 236–241.
137. Bidlack, D.L.; Anderson, D.K. Mutual Diffusion in Nonideal Nonassociating Liquid Systems. *J. Phys. Chem.* **1964**, *68*, 3790–3794.
138. Alizadeh, A.A.; Wakeham, W.A. Mutual Diffusion Coefficients for Binary Mixtures of Normal Alkanes. *Int. J. Thermophys.* **1982**, *3*, 307–323.
139. Padrel de Oliveira, C.M.; Fareleira, J.M.N.A.; Nieto de Castro, C.A. Mutual Diffusivity in Binary Mixtures of N-Heptane with n-Hexane Isomers. *Int. J. Thermophys.* **1989**, *10*, 973–982.
140. Li, S.F.Y.; Yue, L.S. Composition Dependence of Binary Diffusion Coefficients in Alkane Mixtures. *Int. J. Thermophys.* **1990**, *11*, 537–554.
141. Li, S.F.Y.; Wakeham, W.A. Mutual Diffusion Coefficients for Two N-Octane Isomers in n-Heptane. *Int. J. Thermophys.* **1989**, *10*, 995–1003.
142. Matthews, M.A.; Rodden, J.B.; Akgerman, A. High-Temperature Diffusion, Viscosity, and Density Measurements in n-Hexadecane. *J. Chem. Eng. Data* **1987**, *32*, 317–319.
143. Dymond, J.H.; Woolf, L.A. Tracer Diffusion of Organic Solutes in N-Hexane at Pressures up to 400 MPa. *J. Chem. Soc. Faraday Trans. 1* **1982**, *78*, 991–1000.
144. Safi, A.; Nicolas, C.; Neau, E.; Chevalier, J.L. Measurement and Correlation of Diffusion Coefficients of Aromatic Compounds at Infinite Dilution in Alkane and Cycloalkane Solvents. *J. Chem. Eng. Data* **2007**, *52*, 977–981.
145. Leffler, J.; Cullinan, H.T. Variation of Liquid Diffusion Coefficients with Composition. Dilute Ternary Systems. *Ind. Eng. Chem. Fundam.* **1970**, *9*, 88–93.
146. Harris, K.R.; Pua, C.K.N.; Dunlop, P.J. Mutual and Tracer Diffusion Coefficients and Frictional Coefficients for Systems Benzene-Chlorobenzene, Benzene-n-Hexane, and Benzene-n-Heptane at 25 ^{o}C. *J. Phys. Chem.* **1970**, *74*, 3518–3529.
147. Awan, M.A.; Dymond, J.H. Transport Properties of Nonelectrolyte Liquid Mixtures. X. Limiting Mutual Diffusion Coefficients of Fluorinated Benzenes in n-Hexane. *Int. J. Thermophys.* **1996**, *17*, 759–769.
148. Okamoto, M. Diffusion Coefficients Estimated by Dynamic Fluorescence Quenching at High Pressure: Pyrene, 9,10-Dimethylanthracene, and Oxygen in n-Hexane. *Int. J. Thermophys.* **2002**, *23*, 421–435.
149. Bidlack, D.L.; Kett, T.K.; Kelly, C.M.; Anderson, D.K. Diffusion in the Solvents Hexane and Carbon Tetrachloride. *J. Chem. Eng. Data* **1969**, *14*, 342–343.
150. Grushka, E.; Kikta, E.J. Extension of the Chromatographic Broadening Method of Measuring Diffusion Coefficients to Liquid Systems. I. Diffusion Coefficients of Some Alkylbenzenes in Chloroform. *J. Phys. Chem.* **1974**, *78*, 2297–2301.
151. Holmes, J.T.; Olander, D.R.; Wilke, C.R. Diffusion in Mixed Solvents. *AIChE J.* **1962**, *8*, 646–649.
152. Funazukuri, T.; Ishiwata, Y. Diffusion Coefficients of Linoleic Acid Methyl Ester, Vitamin K3 and Indole in Mixtures of Carbon Dioxide and n-Hexane at 313.2 K, and 16.0 MPa and 25.0 MPa. *Fluid Phase Equilib.* **1999**, *164*, 117–129.
153. Fan, Y.Q.; Qian, R.Y.; Shi, M.R.; Shi, J. Infinite Dilution Diffusion Coefficients of Several Aromatic Hydrocarbons in Octane and 2,2,4-Trimethylpentane. *J. Chem. Eng. Data* **1995**, *40*, 1053–1055.
154. Moore, J.W.; Wellek, R.M. Diffusion Coefficients of N-Heptane and n-Decane in n-Alkanes and n-Alcohols at Several Temperatures. *J. Chem. Eng. Data* **1974**, *19*, 136–140.
155. Debenedetti, P.G.; Reid, R.C. Diffusion and Mass Transfer in Supercritical Fluids. *AIChE J.* **1986**, *32*, 2034–2046.
156. Márquez, N.; Kreutzer, M.T.; Makkee, M.; Moulijn, J.A. Infinite Dilution Binary Diffusion Coefficients of Hydrotreating Compounds in Tetradecane in the Temperature Range from (310 to 475) K. *J. Chem. Eng. Data* **2008**, *53*, 439–443.
157. Funazukuri, T.; Kong, C.Y.; Kagei, S. Binary Diffusion Coefficients of Acetone in Carbon Dioxide at 308.2 and 313.2 K in the Pressure Range from 7.9 to 40 MPa. *Int. J. Thermophys.* **2000**, *21*, 651–669.

158. Sassiat, P.R.; Mourier, P.; Caude, M.H.; Rosset, R.H. Measurement of Diffusion Coefficients in Supercritical Carbon Dioxide and Correlation with the Equation of Wilke and Chang. *Anal. Chem.* **1987**, *59*, 1164–1170.
159. Funazukuri, T.; Kong, C.Y.; Kagei, S. Infinite-Dilution Binary Diffusion Coefficients of 2-Propanone, 2-Butanone, 2-Pentanone, and 3-Pentanone in CO_2. *Int. J. Thermophys.* **2000**, *21*, 1279–1290.
160. Kong, C.Y.; Funazukuri, T.; Kagei, S. Chromatographic Impulse Response Technique with Curve Fitting to Measure Binary Diffusion Coefficients and Retention Factors Using Polymer-Coated Capillary Columns. *J. Chromatogr. A* **2004**, *1035*, 177–193.
161. Nishiumi, H.; Fujita, M.; Agou, K. Diffusion of Acetone in Supercritical Carbon Dioxide. *Fluid Phase Equilib.* **1996**, *117*, 356–363.
162. Shenai, V.M.; Hamilton, B.L.; Matthews, M.A. Diffusion in Liquid and Supercritical Fluid Mixtures. In *Supercritical Fluid Engineering Science - Fundamentals and Applications*; Kiran, E., Brennecke, J.F., Eds.; 1992; pp. 92–103.
163. Dahmen, N.; Kordikowski, A.; Schneider, G.M. Determination of Binary Diffusion Coefficients of Organic Compounds in Supercritical Carbon Dioxide by Supercritical Fluid Chromatography. *J. Chromatogr. A* **1990**, *505*, 169–178.
164. Suárez-Iglesias, O.; Medina, I.; Pizarro, C.; Bueno, J.L. Diffusion Coefficients of 2-Fluoroanisole, 2-Bromoanisole, Allylbenzene and 1,3-Divinylbenzene at Infinite Dilution in Supercritical Carbon Dioxide. *Fluid Phase Equilib.* **2007**, *260*, 279–286.
165. González, L.M.; Suárez-Iglesias, O.; Bueno, J.L.; Pizarro, C.; Medina, I. Limiting Binary Diffusivities of Aniline, Styrene, and Mesitylene in Supercritical Carbon Dioxide. *J. Chem. Eng. Data* **2007**, *52*, 1286–1290.
166. González, L.M.; Bueno, J.L.; Medina, I. Determination of Binary Diffusion Coefficients of Anisole, 2,4-Dimethylphenol, and Nitrobenzene in Supercritical Carbon Dioxide. *Ind. Eng. Chem. Res.* **2001**, *40*, 3711–3716.
167. Abaroudi, K. Limpieza de Matrices Sólidas Porosas de Interés Medioambiental Con Fluidos Supercríticos, Universidad Politécnica de Catalunã, 2001.
168. Funazukuri, T.; Kong, C.Y.; Kikuchi, T.; Kagei, S. Measurements of Binary Diffusion Coefficient and Partition Ratio at Infinite Dilution for Linoleic Acid and Arachidonic Acid in Supercritical Carbon Dioxide. *J. Chem. Eng. Data* **2003**, *48*, 684–688.
169. Han, Y.S.; Yang, Y.W.; Wu, P.D. Binary Diffusion Coefficients of Arachidonic Acid Ethyl Ester, Cis-5,8,11,14,17-Eicosapentaenoic Acid Ethyl Ester, and Cis-4,7,10,13,16,19-Docosahexanenoic Acid Ethyl Ester in Supercritical Carbon Dioxide. *J. Chem. Eng. Data* **2007**, *52*, 555–559.
170. Liong, K.K.; Wells, P.A.; Foster, N.R. Diffusion Coefficients of Long-Chain Esters in Supercritical Carbon Dioxide. *Ind. Eng. Chem. Res.* **1991**, *30*, 1329–1335.
171. Filho, C.A.; Silva, C.M.; Quadri, M.B.; Macedo, E.A. Infinite Dilution Diffusion Coefficients of Linalool and Benzene in Supercritical Carbon Dioxide. *J. Chem. Eng. Data* **2002**, *47*, 1351–1354.
172. Swaid, I.; Schneider, G.M. Determination of Binary Diffusion Coefficients of Benzene and Some Alkylbenzenes in Supercritical CO_2 between 308 and 328 K in the Pressure Range 80 to 160 Bar with Supercritical Fluid Chromatography (SFC). *Ber. Bunsenges. Phys. Chem.. Chem.* **1979**, *83*, 969–974.
173. Suárez, J.J.; Bueno, J.L.; Medina, I. Determination of Binary Diffusion Coefficients of Benzene and Derivatives in Supercritical Carbon Dioxide. *Chem. Eng. Sci.* **1993**, *48*, 2419–2427.
174. Funazukuri, T.; Nishimoto, N. Tracer Diffusion Coefficients of Benzene in Dense CO2 at 313.2 K and 8.5-30 MPa. *Fluid Phase Equilib.* **1996**, *125*, 235–243.
175. Funazukuri, T.; Kong, C.Y.; Kagei, S. Infinite Dilution Binary Diffusion Coefficients of Benzene in Carbon Dioxide by the Taylor Dispersion Technique at Temperatures from 308.15 to 328.15 K and Pressures from 6 to 30 MPa. *Int. J. Thermophys.* **2001**, *22*, 1643–1660.
176. Sengers, J.M.H.L.; Deiters, U.K.; Klask, U.; Swidersky, P.; Schneider, G.M. Application of the Taylor Dispersion Method in Supercritical Fluids. *Int. J. Thermophys.* **1993**, *14*, 893–922.
177. Ago, K.; Nishiumi, H. Mutual Diffusion Coefficients of Benzene in Supercritical Carbon Dioxide. *J. Chem. Eng. Japan* **1999**, *32*, 563–568.
178. Bueno, J.L.; Suárez, J.J.; Dizy, J.; Medina, I. Infinite Dilution Diffusion Coefficients:Benzene Derivatives as Solutes in Supercritical Carbon Dioxide. *J. Chem. Eng. Data* **1993**, *38*, 344–349.
179. Kong, C.Y.; Funazukuri, T.; Kagei, S. Binary Diffusion Coefficients and Retention Factors for Polar Compounds in Supercritical Carbon Dioxide by Chromatographic Impulse Response Method. *J. Supercrit. Fluids* **2006**, *37*, 359–366.
180. Catchpole, O.J.; King, M.B. Measurement and Correlation of Binary Diffusion Coefficients in near Critical Fluids. *Ind. Eng. Chem. Res.* **1994**, *33*, 1828–1837.
181. Fu, H.; Coelho, L.A.F.; Matthews, M.A. Diffusion Coefficients of Model Contaminants in Dense CO<sub>2<\sub>. *J. Supercrit. Fluids* **2000**, *18*, 141–155.

182. Suárez-Iglesias, O.; Medina, I.; Pizarro, C.; Bueno, J.L. Limiting Diffusion Coefficients of Ethyl Benzoate, Benzylacetone, and Eugenol in Carbon Dioxide at Supercritical Conditions. *J. Chem. Eng. Data* **2008**, *53*, 779–784.
183. González, L.M.; Suárez-Iglesias, O.; Bueno, J.L.; Pizarro, C.; Medina, I. Application of the Corresponding States Principle to the Diffusion in CO2. *AIChE J.* **2007**, *53*, 3054–3061.
184. Suárez, J.J.; Medina, I.; Bueno, J.L. Diffusion Coefficients in Supercritical Fluids: Available Data and Graphical Correlations. *Fluid Phase Equilib.* **1998**, *153*, 167–212.
185. Funazukuri, T. Measurements of Binary Diffusion Coefficients of 20 Organic Compounds in CO_2 at 313.2 K and 16.0 MPa. *J. Chem. Eng. Japan* **1996**, *29*, 191–192.
186. Pizarro, C.; Suárez-Iglesias, O.; Medina, I.; Bueno, J.L. Diffusion Coefficients of N-Butylbenzene, n-Pentylbenzene, 1-Phenylhexane, 1-Phenyloctane, and 1-Phenyldodecane in Supercritical Carbon Dioxide. *Ind. Eng. Chem. Res.* **2008**, *47*, 6783–6789.
187. González, L.M.; Bueno, J.L.; Medina, I. Measurement of Diffusion Coefficients for 2-Nitroanisole, 1,2-Dichlorobenzene and Tert-Butylbenzene in Carbon Dioxide Containing Modifiers. *J. Supercrit. Fluids* **2002**, *24*, 219–229.
188. Liong, K.K.; Wells, P.A.; Foster, N.R. Diffusion of Fatty Acid Esters in Supercritical Carbon Dioxide. *Ind. Eng. Chem. Res.* **1992**, *31*, 390–399.
189. Wells, P.A. Diffusion in Supercritical Fluids, The University of New South Wales, Kensington, Australia, 1991.
190. Lai, C.-C.; Tan, C.-S. Measurement of Molecular Diffusion Coefficient in Supercritical Carbon Dioxide Using a Coated Capillary Column. *Ind. Eng. Chem. Res.* **1995**, *34*, 674–680.
191. Knaff, G.; Schlünder, E.U. Diffusion Coefficients of Naphthalene and Caffeine in Supercritical Carbon Dioxide. *Chem. Eng. Process.* **1987**, *21*, 101–105.
192. Lauer, H.H.; McManigill, D.; Board, R.D. Mobile-Phase Transport Properties of Liquefied Gases in near Critical and Supercritical Fluid Chromatography. *Anal. Chem.* **1983**, *55*, 1370–1375.
193. Funazukuri, T.; Kong, C.Y.; Kagei, S. Binary Diffusion Coefficients, Partition Ratios and Partial Molar Volumes at Infinite Dilution for β-Carotene and α-Tocopherol in Supercritical Carbon Dioxide. *J. Supercrit. Fluids* **2003**, *27*, 85–96.
194. Funazukuri, T.; Kong, C.Y.; Murooka, N.; Kagei, S. Measurements of Binary Diffusion Coefficients and Partition Ratios for Acetone, Phenol, α-Tocopherol, and β-Carotene in Supercritical Carbon Dioxide with a Poly(Ethylene Glycol)-Coated Capillary Column. *Ind. Eng. Chem. Res.* **2000**, *39*, 4462–4469.
195. Funazukuri, T.; Kong, C.Y.; Kagei, S. Measurements of Binary Diffusion Coefficients for Some Low Volatile Compounds in Supercritical Carbon Dioxide by Input–Output Response Technique with Two Diffusion Columns Connected in Series. *Fluid Phase Equilib.* **2002**, *194*, 1169–1178.
196. Dong, X.; Su, B.; Xing, H.; Bao, Z.; Yang, Y.; Ren, Q. Cosolvent Effects on the Diffusions of 1,3-Dichlorobenzene, L-Carvone, Geraniol and 3-Fluorophenol in Supercritical Carbon Dioxide. **2011**, *58*, 216–225.
197. Dong, X.; Su, B.; Xing, H.; Yang, Y.; Ren, Q. Diffusion Coefficients of L-Menthone and L-Carvone in Mixtures of Carbon Dioxide and Ethanol. *J. Supercrit. Fluids* **2010**, *55*, 86–95.
198. Cordeiro, J.; Magalhães, A.L.; Valente, A.A.; Silva, C.M. Experimental and Theoretical Analysis of the Diffusion Behavior of Chromium(III) Acetylacetonate in Supercritical CO_2. *J. Supercrit. Fluids* **2016**, *118*, 153–162.
199. Filho, C.A.; Silva, C.M.; Quadri, M.B.; Macedo, E.A. Tracer Diffusion Coefficients of Citral and D-Limonene in Supercritical Carbon Dioxide. *Fluid Phase Equilib.* **2003**, *204*, 65–73.
200. Kong, C.Y.; Gu, Y.Y.; Nakamura, M.; Funazukuri, T.; Kagei, S. Diffusion Coefficients of Metal Acetylacetonates in Supercritical Carbon Dioxide. *Fluid Phase Equilib.* **2010**, *297*, 162–167.
201. Yang, Y.N.; Matthews, M.A. Diffusion of Chelating Agents in Supercritical CO2 and a Predictive Approach for Diffusion Coefficients. *J. Chem. Eng. Data* **2001**, *46*, 588–595.
202. Kong, C.Y.; Takahashi, N.; Funazukuri, T.; Kagei, S. Measurements of Binary Diffusion Coefficients and Retention Factors for Dibenzo-24-Crown-8 and 15-Crown-5 in Supercritical Carbon Dioxide by Chromatographic Impulse Response Technique. *Fluid Phase Equilib.* **2007**, *257*, 223–227.
203. Dahmen, N.; Dulberg, A.; Schneider, G.M.M. Determination of Binary Diffusion Coefficients in Supercritical Carbon Dioxide with Supercritical Fluid Chromatography (SFC). *Ber. Bunsenges. Phys. Chem.* **1990**, *94*, 384–386.
204. Umezawa, S.; Nagashima, A. Measurement of the Diffusion Coefficients of Acetone, Benzene, and Alkane in Supercritical CO2 by the Taylor Dispersion Method. *J. Supercrit. Fluids* **1992**, *5*, 242–250.
205. Silva, C.M.; Macedo, E.A. Diffusion Coefficients of Ethers in Supercritical Carbon Dioxide. *Ind. Eng. Chem. Res.* **1998**, *37*, 1490–1498.

206. Pizarro, C.; Suárez-Iglesias, O.; Medina, I.; Bueno, J.L. Using Supercritical Fluid Chromatography to Determine Diffusion Coefficients of 1,2-Diethylbenzene, 1,4-Diethylbenzene, 5-Tert-Butyl-m-Xylene and Phenylacetylene in Supercritical Carbon Dioxide. *J. Chromatogr. A* **2007**, *1167*, 202–209.
207. Pizarro, C.; Suárez-Iglesias, O.; Medina, I.; Bueno, J.L. Binary Diffusion Coefficients for 2,3-Dimethylaniline, 2,6-Dimethylaniline, 2-Methylanisole, 4-Methylanisole and 3-Nitrotoluene in Supercritical Carbon Dioxide. *J. Supercrit. Fluids* **2009**, *48*, 1–8.
208. Kong, C.Y.; Nakamura, M.; Sone, K.; Funazukuri, T.; Kagei, S. Measurements of Binary Diffusion Coefficients for Ferrocene and 1,1′-Dimethylferrocene in Supercritical Carbon Dioxide. *J. Chem. Eng. Data* **2010**, *55*, 3095–3100.
209. Higashi, H.; Iwai, Y.; Nakamura, Y.; Yamamoto, S.; Arai, Y. Correlation of Diffusion Coefficients for Naphthalene and Dimethylnaphthalene Isomers in Supercritical Carbon Dioxide. *Fluid Phase Equilib.* **1999**, *166*, 101–110.
210. Higashi, H.; Iwai, Y.; Takahashi, Y.; Uchida, H.; Arai, Y. Diffusion Coefficients of Naphthalene and Dimethylnaphthalene in Supercritical Carbon Dioxide. *Fluid Phase Equilib.* **1998**, *144*, 269–278.
211. Funazukuri, T.; Kong, C.Y.; Kagei, S. Effects of Molecular Weight and Degree of Unsaturation on Binary Diffusion Coefficients for Lipids in Supercritical Carbon Dioxide. *Fluid Phase Equilib.* **2004**, *219*, 67–73.
212. Funazukuri, T.; Kong, C.Y.; Kagei, S. Binary Diffusion Coefficient, Partition Ratio, and Partial Molar Volume for Docosahexaenoic Acid, Eicosapentaenoic Acid and α-Linolenic Acid at Infinite Dilution in Supercritical Carbon Dioxide. *Fluid Phase Equilib.* **2003**, *206*, 163–178.
213. Pizarro, C.; Suárez-Iglesias, O.; Medina, I.; Bueno, J.L. Binary Diffusion Coefficients of 2-Ethyltoluene, 3-Ethyltoluene, and 4-Ethyltoluene in Supercritical Carbon Dioxide. *J. Chem. Eng. Data* **2009**, *54*, 1467–1471.
214. Kong, C.Y.; Sone, K.; Sako, T.; Funazukuri, T.; Kagei, S. Solubility Determination of Organometallic Complexes in Supercritical Carbon Dioxide by Chromatographic Impulse Response Method. *Fluid Phase Equilib.* **2011**, *302*, 347–353.
215. Akgerman, A.; Erkey, C.; Orejuela, M. Limiting Diffusion Coefficients of Heavy Molecular Weight Organic Contaminants in Supercritical Carbon Dioxide. *Ind. Eng. Chem. Res.* **1996**, *35*, 911–917.
216. Lin, R.; Tavlarides, L.L. Diffusion Coefficients of Diesel Fuel and Surrogate Compounds in Supercritical Carbon Dioxide. *J. Supercrit. Fluids* **2010**, *52*, 47–55.
217. Funazukuri, T.; Hachisu, S.; Wakao, N. Measurements of Binary Diffusion Coefficients of C16-C24 Unsaturated Fatty Acid Methyl Esters in Supercritical Carbon Dioxide. *Ind. Eng. Chem. Res.* **1991**, *30*, 1323–1329.
218. Kong, C.Y.; Withanage, N.R.W.; Funazukuri, T.; Kagei, S. Binary Diffusion Coefficients and Retention Factors for γ-Linolenic Acid and Its Methyl and Ethyl Esters in Supercritical Carbon Dioxide. *J. Supercrit. Fluids* **2006**, *37*, 63–71.
219. Kong, C.Y.; Mori, M.; Funazukuri, T.; Kagei, S. Measurements of Binary Diffusion Coefficients, Retention Factors and Partial Molar Volumes for Myristoleic Acid and Its Methyl Ester in Supercritical Carbon Dioxide. *Anal. Sci.* **2006**, *22*, 1431–1436.
220. Funazukuri, T.; Ishiwata, Y.; Wakao, N. Predictive Correlation for Binary Diffusion Coefficients in Dense Carbon Dioxide. *AIChE J.* **1992**, *38*, 1761–1768.
221. Lamb, D.M.; Adamy, S.T.; Woo, K.W.; Jonas, J. Transport and Relaxation of Naphthalene in Supercritical Fluids. *J. Phys. Chem.* **1989**, *93*, 5002–5005.
222. Funazukuri, T.; Hachisu, S.; Wakao, N. Measurement of Diffusion Coefficients of C18 Unsaturated Fatty Acid Methyl Esters, Naphthalene, and Benzene in Supercritical Carbon Dioxide by a Tracer Response Technique. *Anal. Chem.* **1989**, *61*, 118–122.
223. Wells, T.; Foster, N.R.; Chaplin, R.P. Diffusion of Phenylacetic Acid and Vanillin in Supercritical Carbon Dioxide. *Ind. Eng. Chem. Res.* **1992**, *31*, 927–934.
224. Pizarro, C.; Suárez-Iglesias, O.; Medina, I.; Bueno, J.L. Molecular Diffusion Coefficients of Phenylmethanol, 1-Phenylethanol, 2-Phenylethanol, 2-Phenyl-1-Propanol, and 3-Phenyl-1-Propanol in Supercritical Carbon Dioxide. *J. Supercrit. Fluids* **2008**, *43*, 469–476.
225. Silva, C.M.; Filho, C.A.; Quadri, M.B.; Macedo, E.A. Binary Diffusion Coefficients of α-Pinene and β-Pinene in Supercritical Carbon Dioxide. *J. Supercrit. Fluids* **2004**, *32*, 167–175.
226. Vaz, R. V.; Magalhães, A.L.; Valente, A.A.; Silva, C.M. Measurement and Modeling of Tracer Diffusivities of α-Pinene in Supercritical CO_2, and Analysis of Their Hydrodynamic and Free-Volume Behaviors. *J. Supercrit. Fluids* **2016**, *107*, 690–698.
227. Suárez, J.J.; Bueno, J.L.; Medina, I.; Dizy, J. Applications of supercritical chromatography - Determination of molecular diffusivity. *Afinidad* **1992**, *49*, 101–113.

228. Kong, C.Y.; Withanage, N.R.W.; Funazukuri, T.; Kagei, S. Binary Diffusion Coefficients and Retention Factors for Long-Chain Triglycerides in Supercritical Carbon Dioxide by the Chromatographic Impulse Response Method. *J. Chem. Eng. Data* **2005**, *50*, 1635–1640.
229. Funazukuri, T.; Kong, C.Y.; Kagei, S. Infinite-Dilution Binary Diffusion Coefficient, Partition Ratio, and Partial Molar Volume for Ubiquinone CoQ10 in Supercritical Carbon Dioxide. *Ind. Eng. Chem. Res.* **2002**, *41*, 2812–2818.
230. Xu, B.; Nagashima, K.; DeSimone, J.M.; Johnson Jr., C.S. Diffusion of Water in Liquid and Supercritical Carbon Dioxide: An NMR Study. *J. Phys. Chem. A* **2003**, *107*, 1–3.
